# Supplementary figures and images for: Intact HIV DNA decays in children with and without complete viral load suppression
Source: PLoS Pathog. 2025 Apr 4;21(4):e1013003. doi: 10.1371/journal.ppat.1013003 (PMC12002518; doi:10.1371/journal.ppat.1013003)

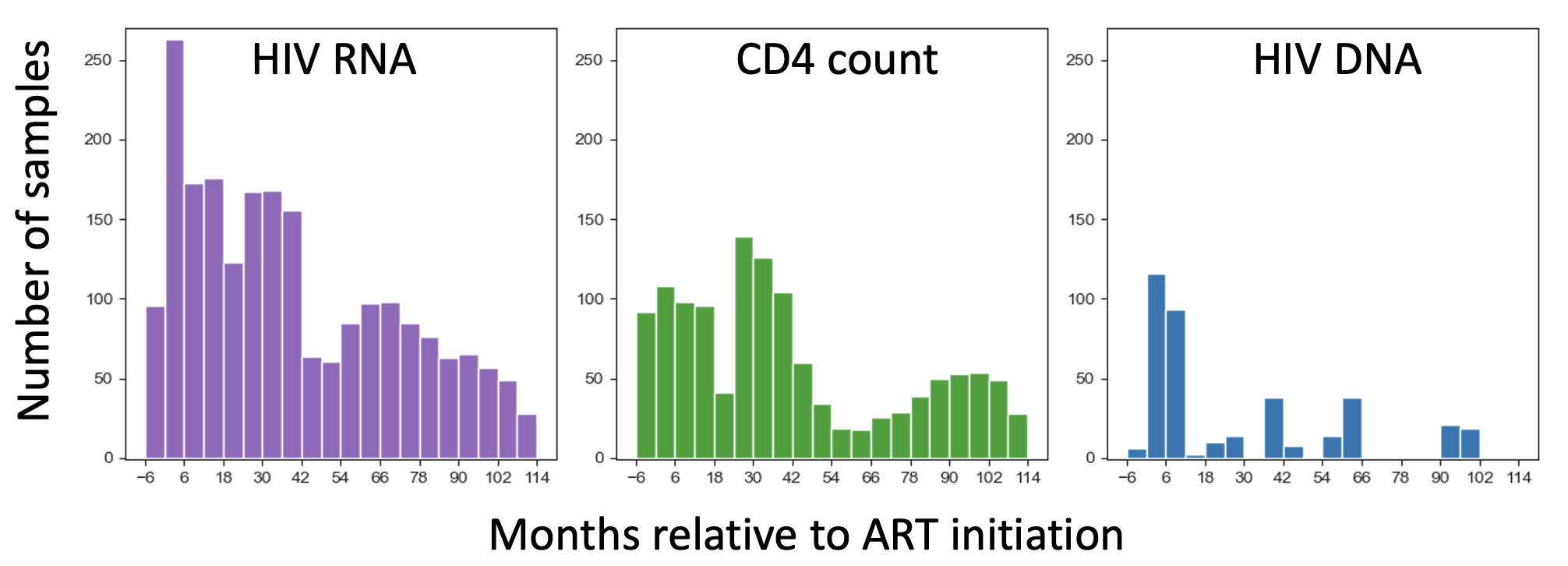

Supplement: S1 Fig — (TIF) [file ppat.1013003.s002.tif]

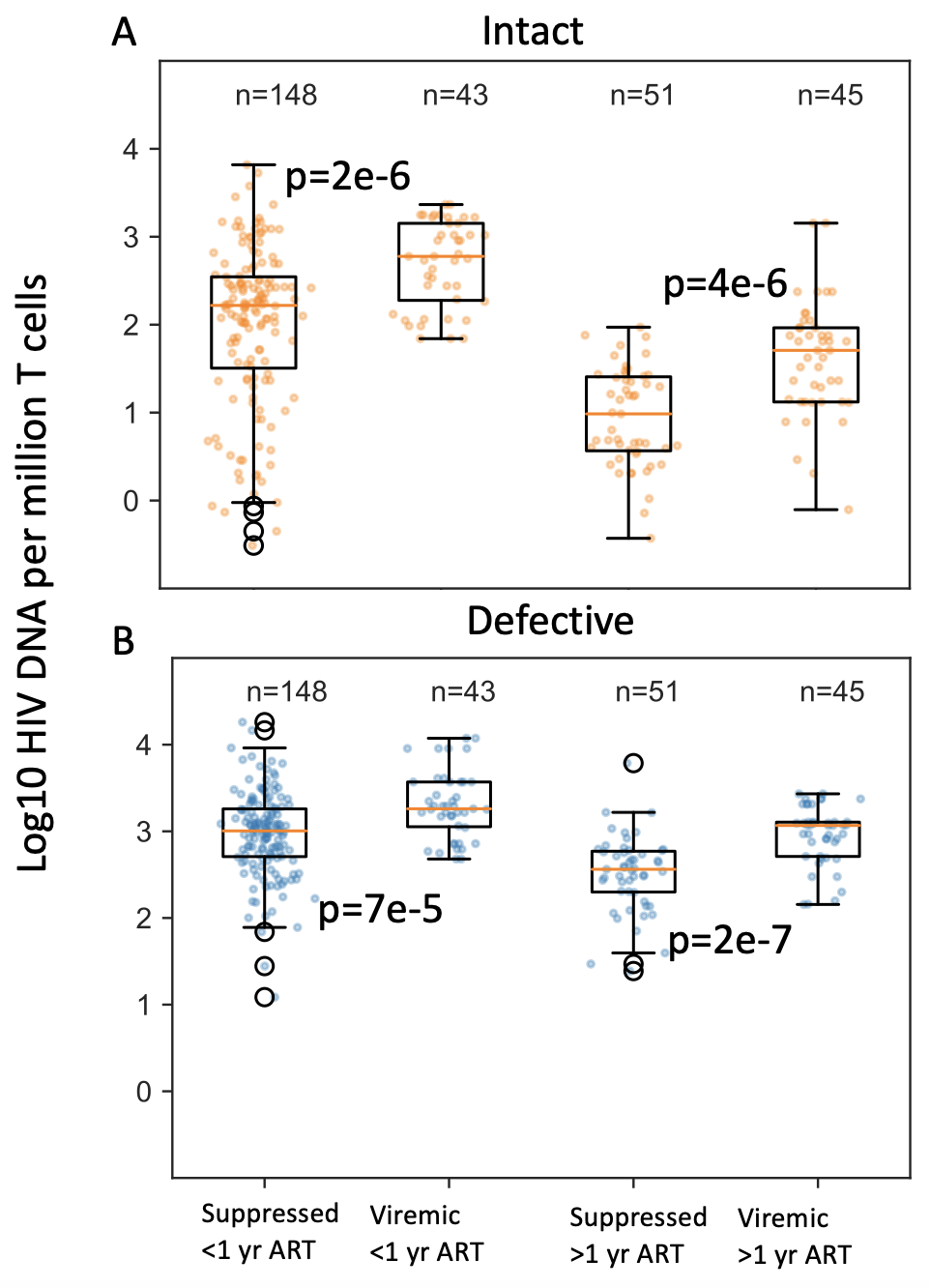

Supplement: S2 Fig — A) Intact and B) defective HIV DNA levels in the suppressed vs viremic subsets (suppressed criteria was HIV RNA levels dropping below 1000 copies/mL within 1 year of starting ART and remaining below 3000 copies/mL thereafter). n indicates number of time points in each boxplot. P-values are from one-sided Mann-Whitney U test comparing the boxes with the horizontal line. Box plots show medians, interquartile ranges and 1.5x interquartile ranges. (TIF) [file ppat.1013003.s003.tif]

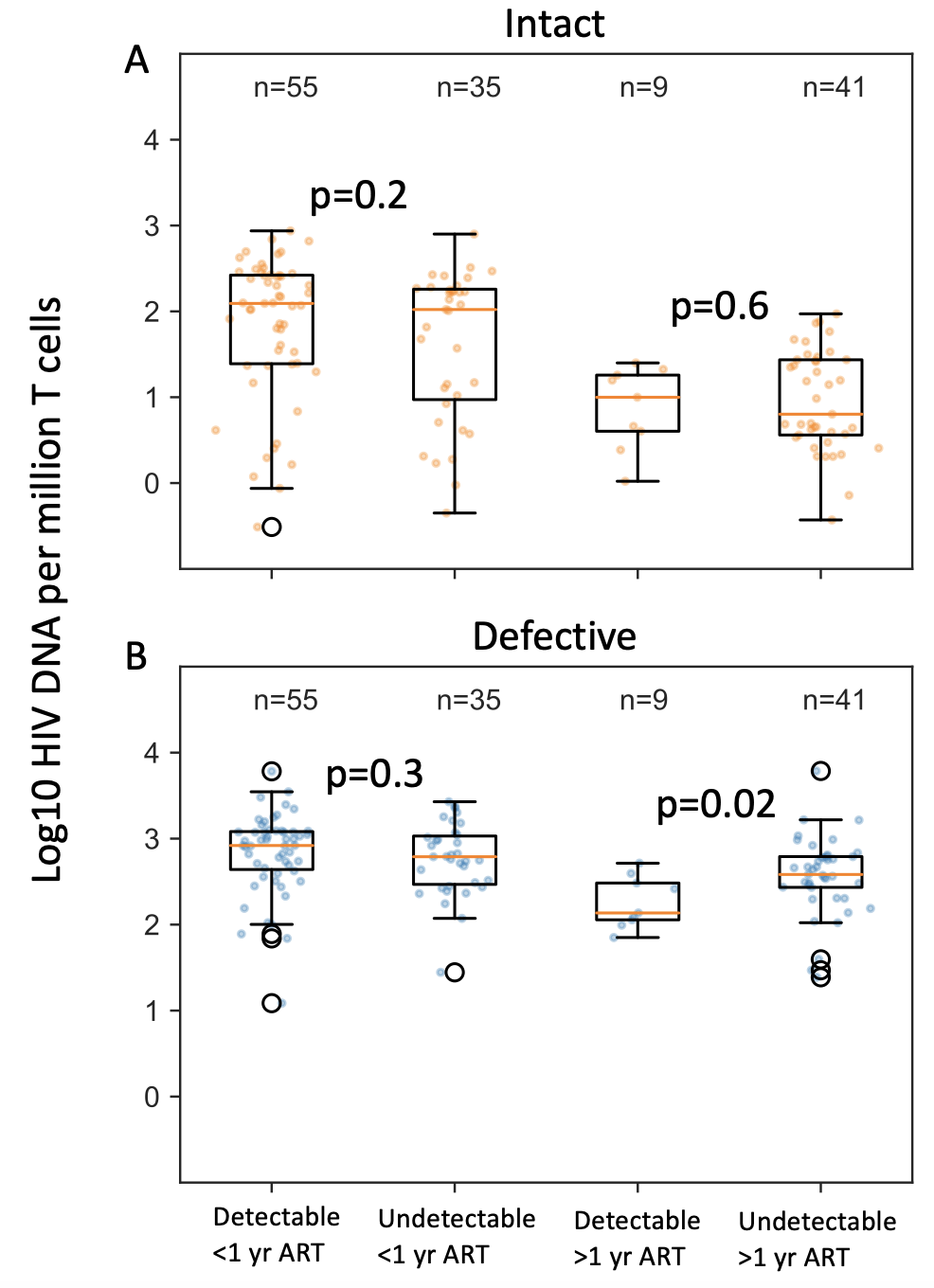

Supplement: S3 Fig — n indicates number of time points in each box. There was no difference between intact and defective HIV DNA levels in participants with low but detectable HIV RNA vs completely undetectable HIV RNA. P-values are Mann-Whitney indicating differences between the two boxes with the horizontal line. Box plots show medians, interquartile ranges and 1.5x interquartile ranges. (TIF) [file ppat.1013003.s004.tif]

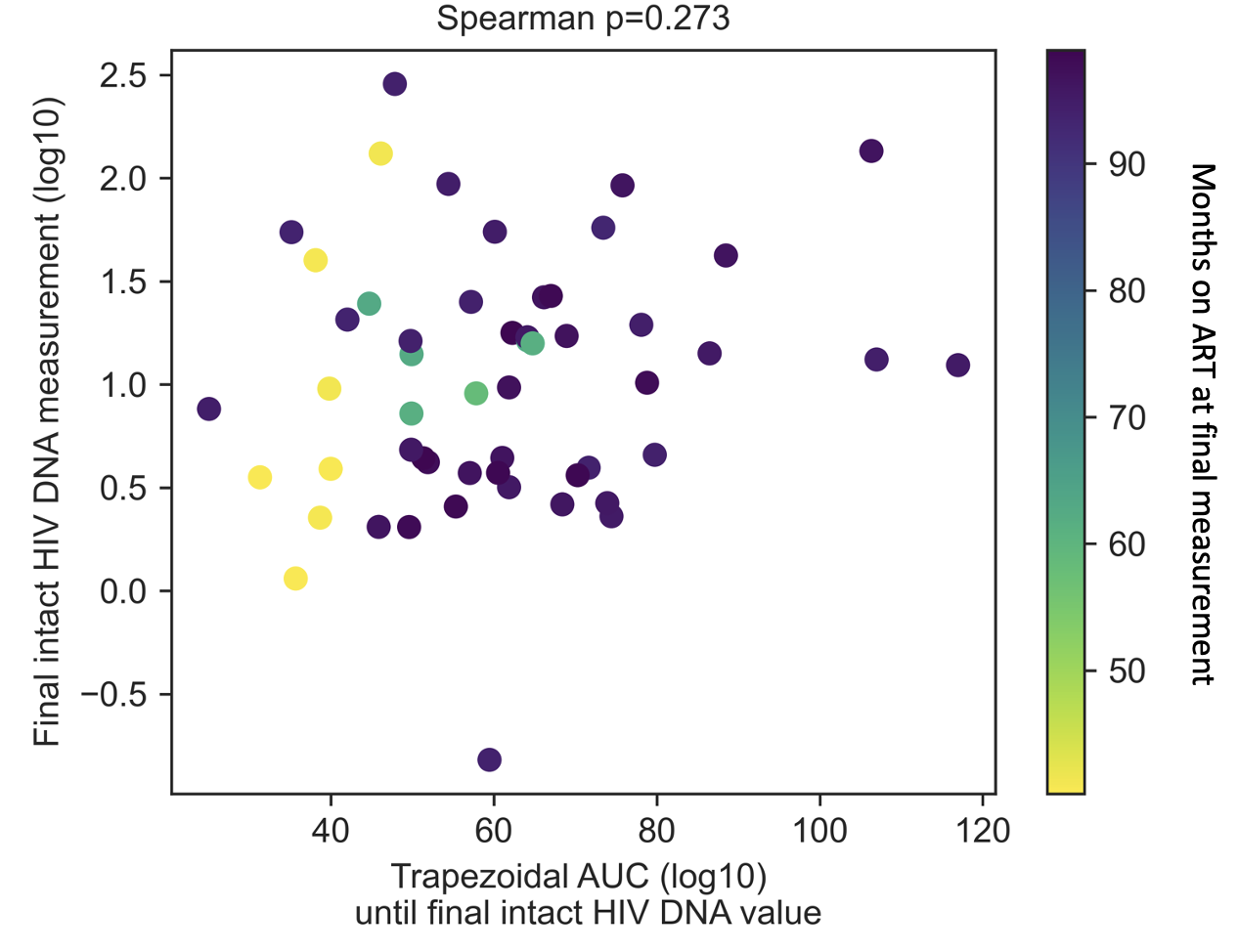

Supplement: S4 Fig — We use a trapezoidal numerical integration to estimate the AUC up until the time of the final intact HIV DNA measurement and then compare this to the final measurement. The dot color indicates the final time point, so darker points are later in time and thus have higher AUC. But, there is not a strong statistical relationship, p=0.3 noted in panel title, and note p=0.9 for the identical analysis for defective HIV DNA. (TIF) [file ppat.1013003.s005.tif]

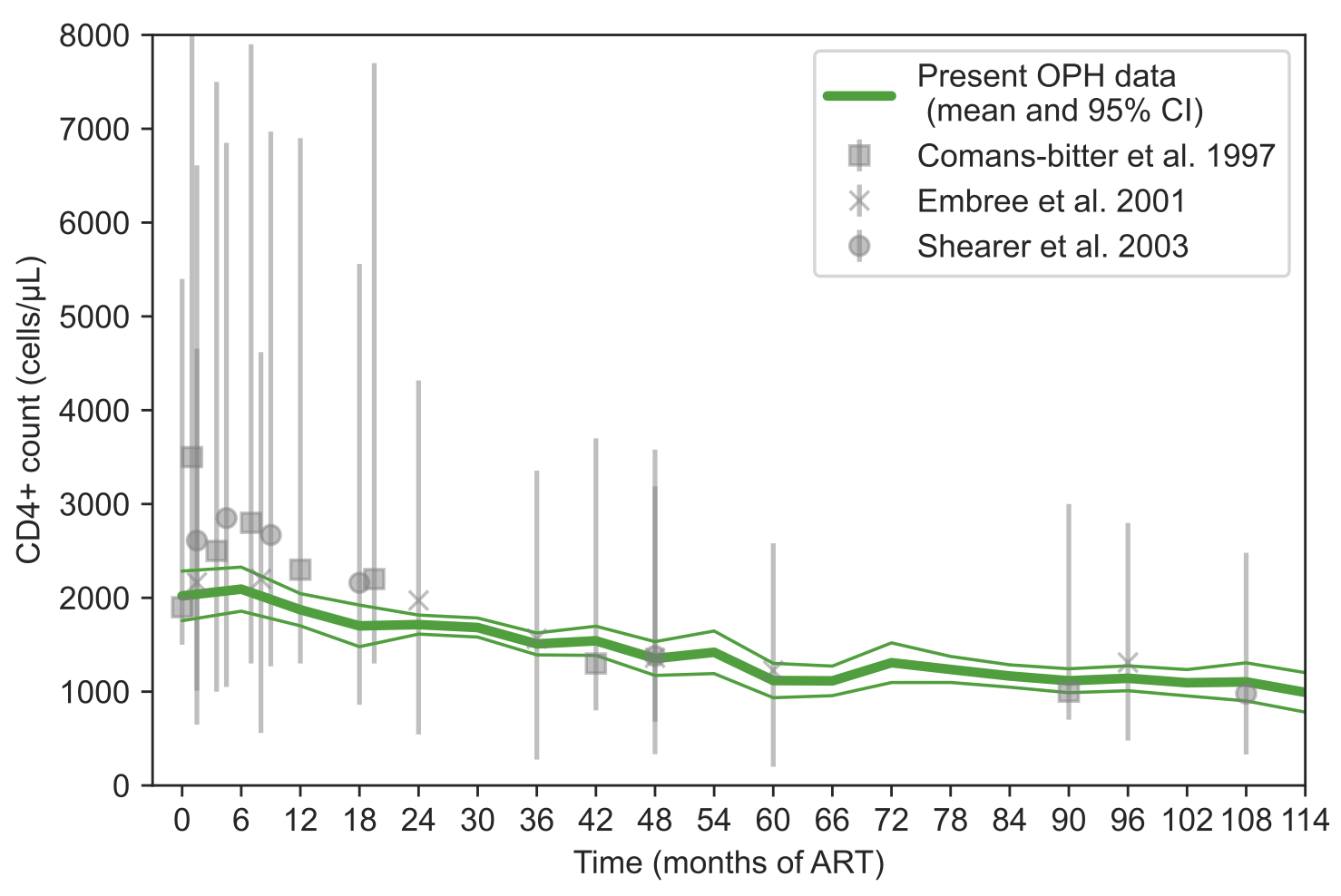

Supplement: S5 Fig — The studies are denoted by the marker, means are represented by dots and vertical lines are 95% confidence intervals as reported in the original studies. The thicker green line indicates the mean value averaged in 6 month windows for the OPH participants, and the thin green lines represent 95% confidence intervals on these estimates. In both OPH participants and historic controls there appears to be a slight rise in the first year of life and a gradual decay afterwards. (TIF) [file ppat.1013003.s006.tif]

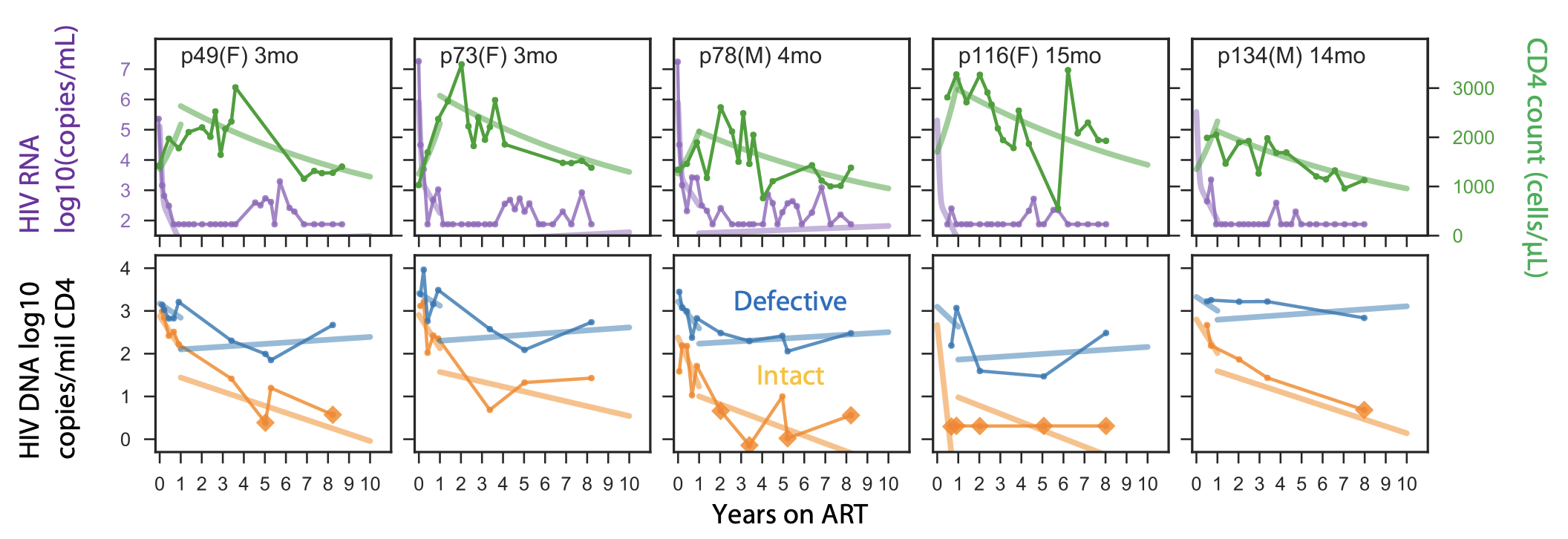

Supplement: S6 Fig — Top row axes indicate HIV RNA (left) and CD4 count (right). Jagged lines with small dots are observed data, solid lines are best model fits. Diamonds for intact HIV DNA indicate levels below limit of detection. The population trends hold for individuals. (TIF) [file ppat.1013003.s007.tif]

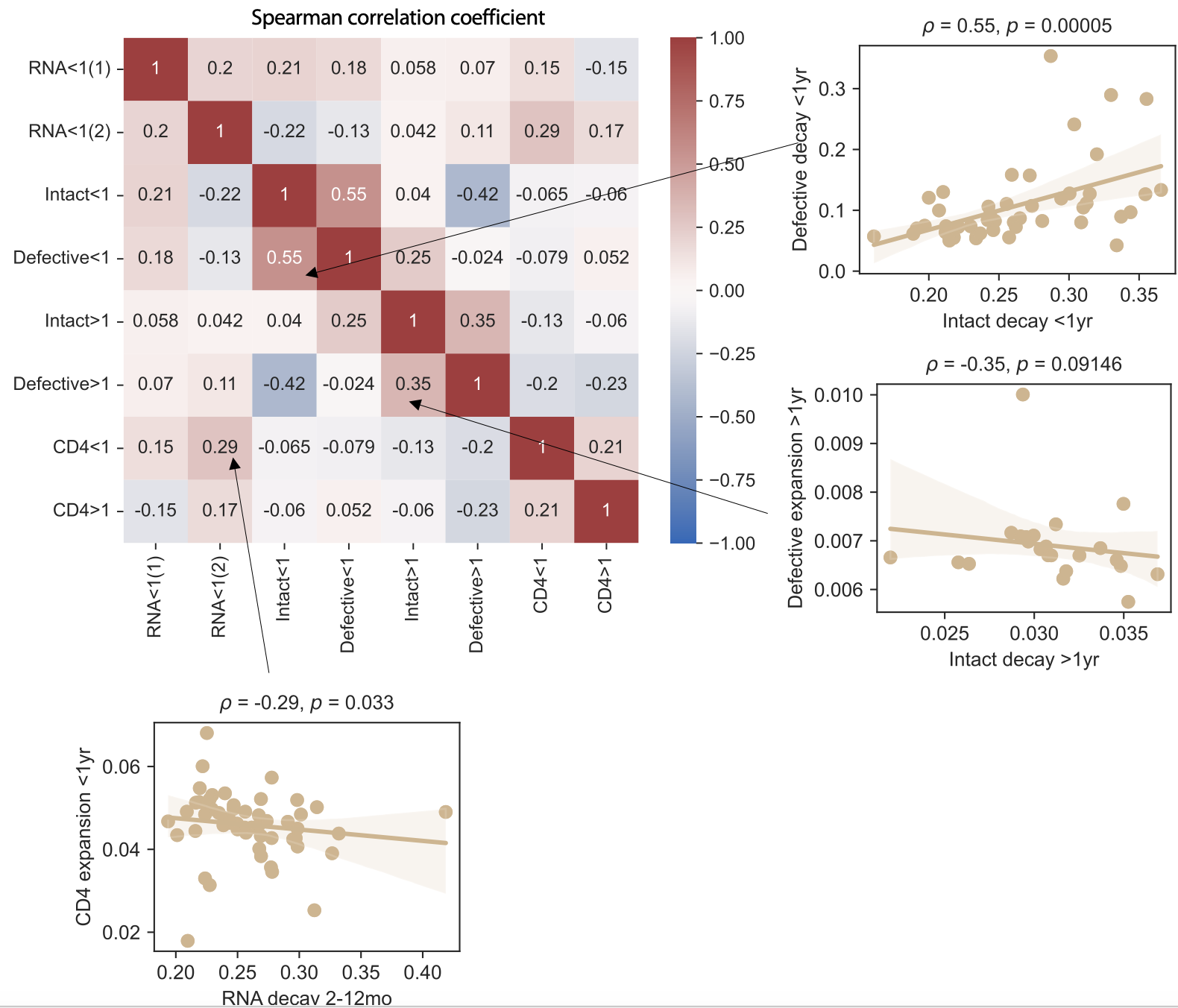

Supplement: S7 Fig — (TIF) [file ppat.1013003.s008.tif]

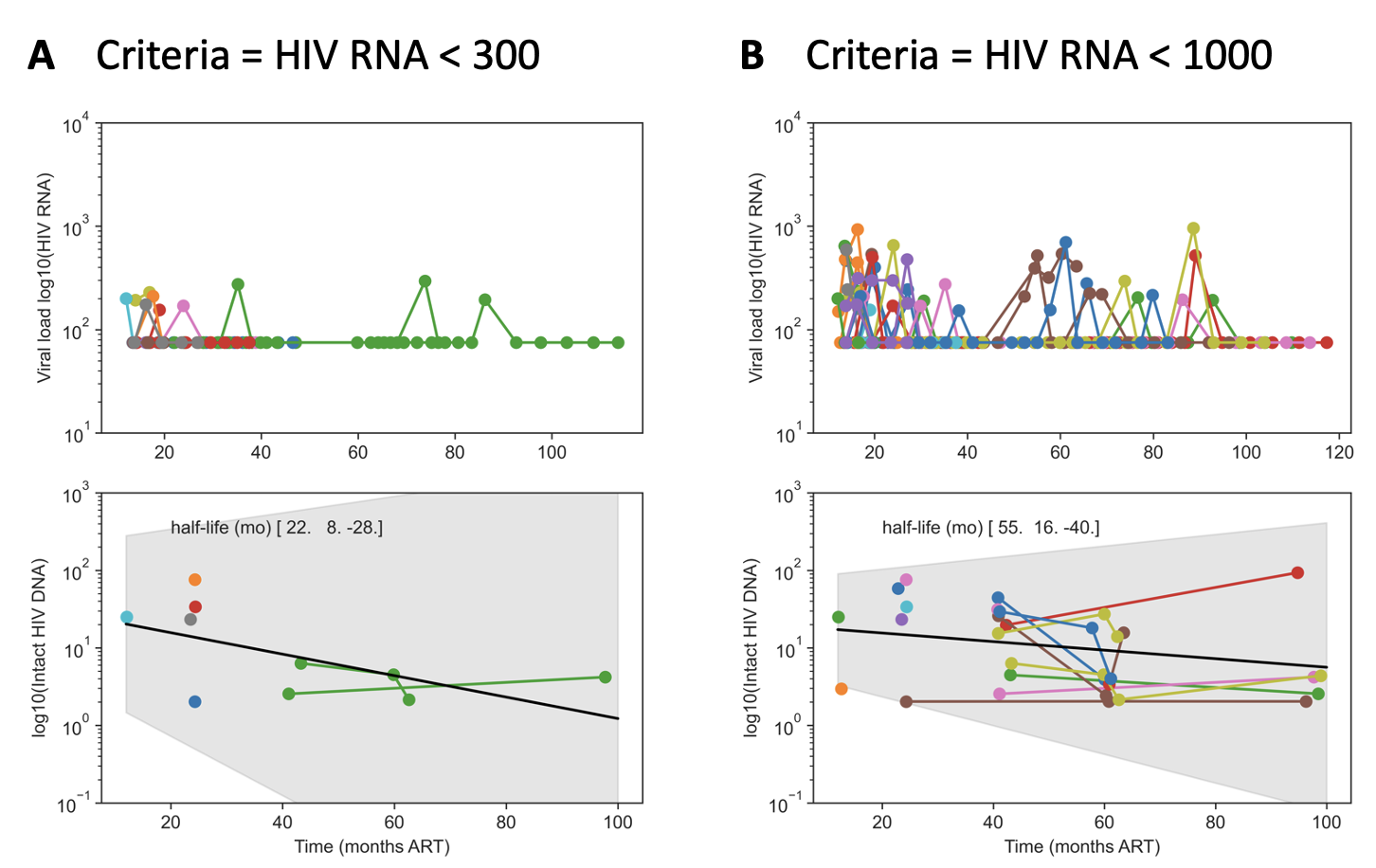

Supplement: S8 Fig — A) Modeling data from participants who had HIV RNA levels below 300 copies/mL at observed time points after 1 year on ART. Left) Viral load levels. Right) Intact HIV DNA levels (colored dots/lines for each participant, matching VL) and mixed effects model decay estimate (black line = population mean, shaded gray = confidence intervals, the values are noted in brackets as [mean, lower 95% CI, upper 95%CI]. There were 7 children who had any intact time points given this criteria and only 2 child with more than 1 longitudinal time point. B) Same plots but HIV RNA < 1000 copies/mL. There were 17 children who had any intact time points given this criteria and 9 children with more than 1 longitudinal time point. (TIF) [file ppat.1013003.s009.tif]

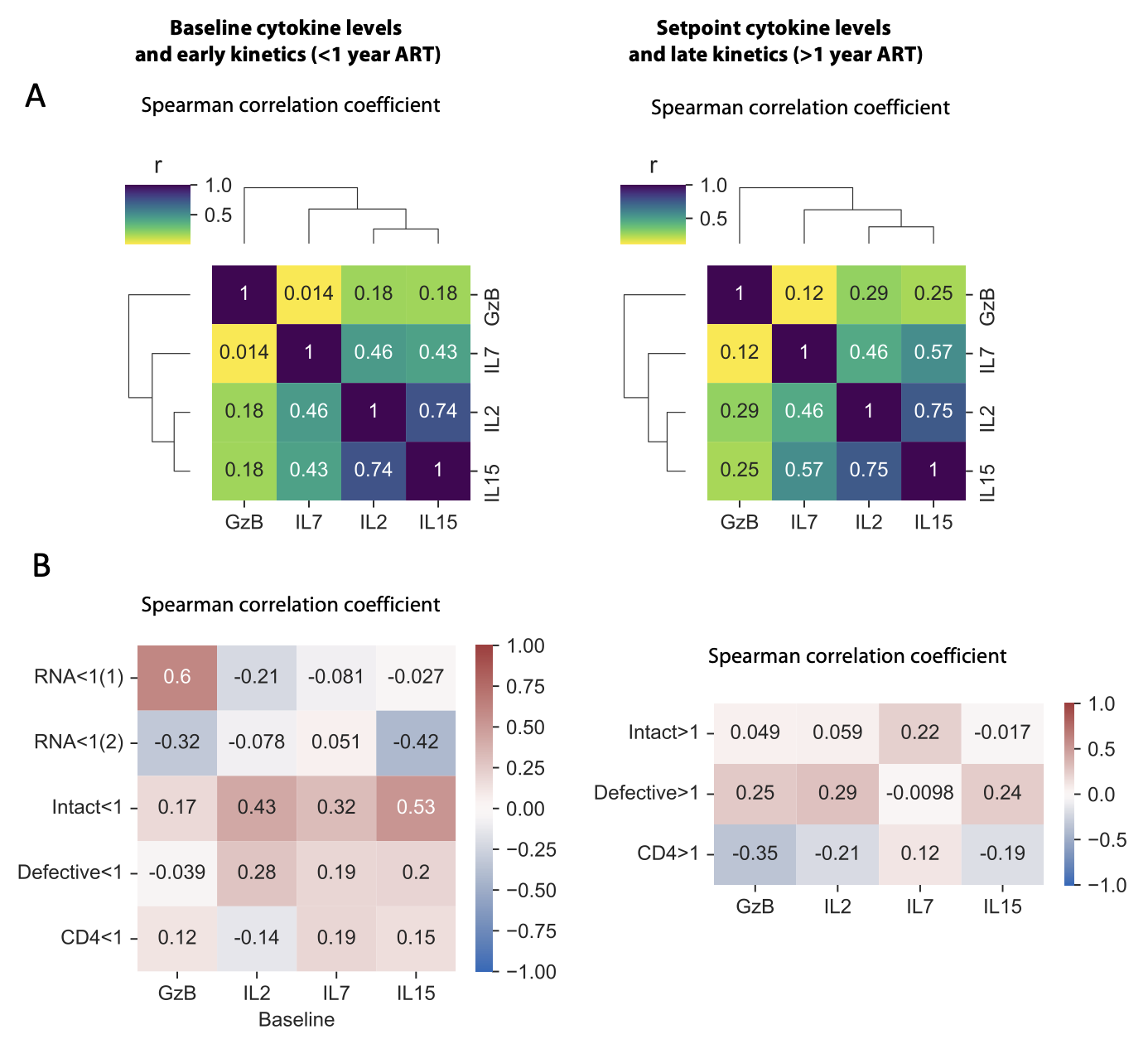

Supplement: S9 Fig — A) Spearman correlation between 4 immune marker levels at baseline (at ART initiation) and during setpoint (average >1yr of ART). P values are noted for each correlation coefficient. B) Spearman correlation coefficient between immune marker levels and HIV RNA, DNA and CD4+ T cell kinetic rates estimated via pNLME models for 18 participants who had rate estimates for all data types. Baseline immune marker levels are compared against rates estimated from early (<1 yr) of ART and setpoint levels are compared against rates from long-term (>1 yr) ART. P values are noted for each correlation coefficient. (TIF) [file ppat.1013003.s010.tif]

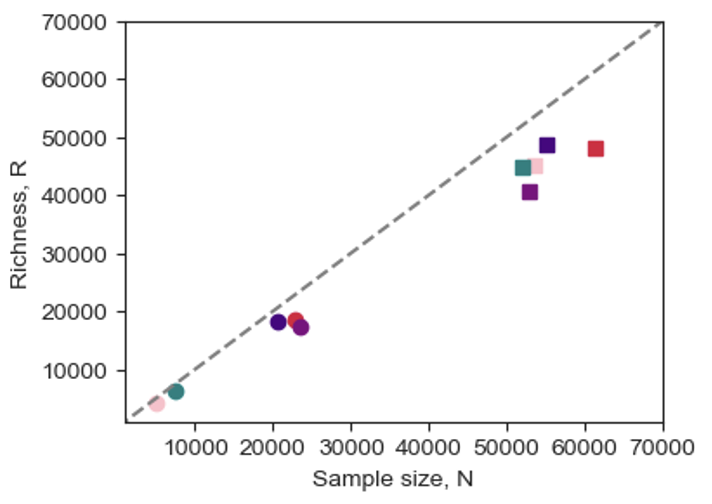

Supplement: S10 Fig — (TIF) [file ppat.1013003.s011.tif]
