# Supplementary material for: Intact HIV DNA decays in children with and without complete viral load suppression
Source: PLoS Pathog. 2025 Apr 4;21(4):e1013003. doi: 10.1371/journal.ppat.1013003 (PMC12002518; doi:10.1371/journal.ppat.1013003)
Supplement: S1 Data — (PDF) [file ppat.1013003.s012.pdf]

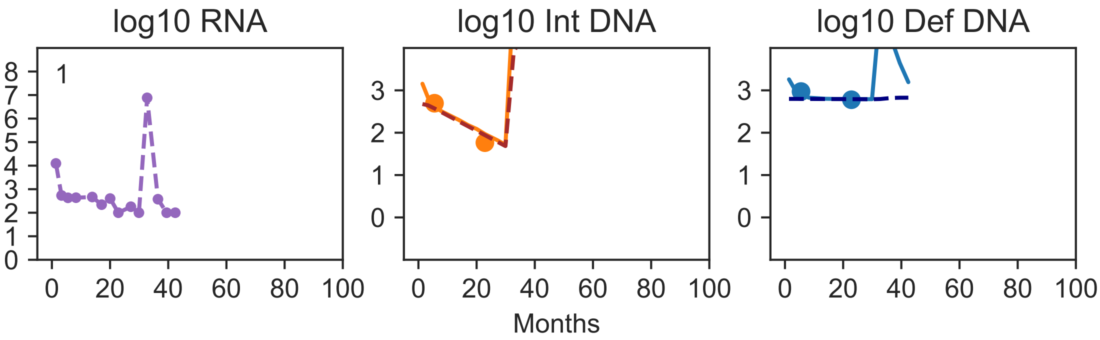

log10 RNA
log10 Int DNA
log10 Def DNA
1
8
7
6
5
4
3
2
1
0
3
2
1
0
3
2
1
0
0
20
40
60
80
100
0
20
40
60
80
100
0
20
40
60
80
100
Months

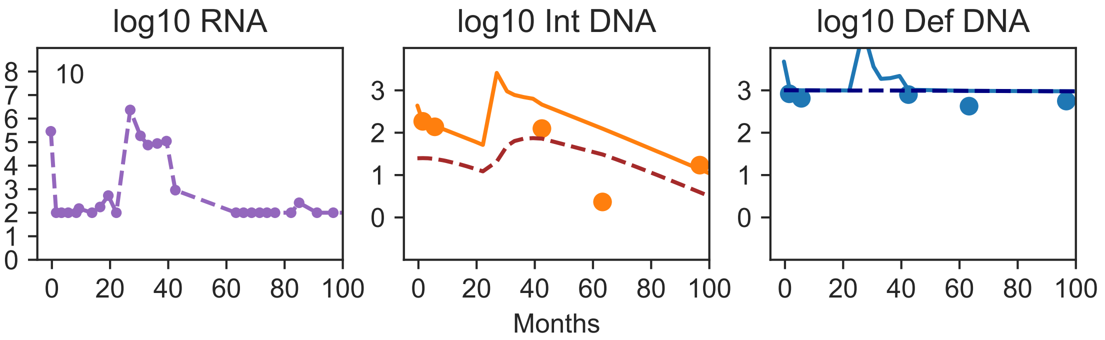

log10 RNA
log10 Int DNA
log10 Def DNA
10
0
1
2
3
4
5
6
7
8
0
20
40
60
80
100
Months

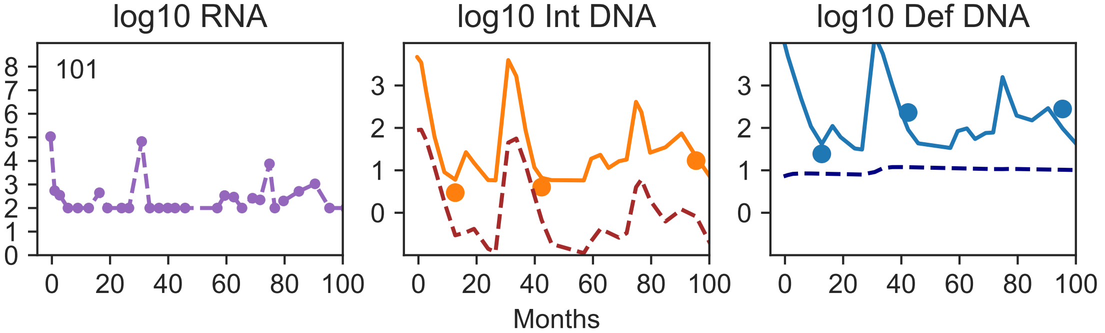
log10 RNA
log10 Int DNA
log10 Def DNA
101
Months
0
1
2
3
4
5
6
7
8
20
40
60
80
100

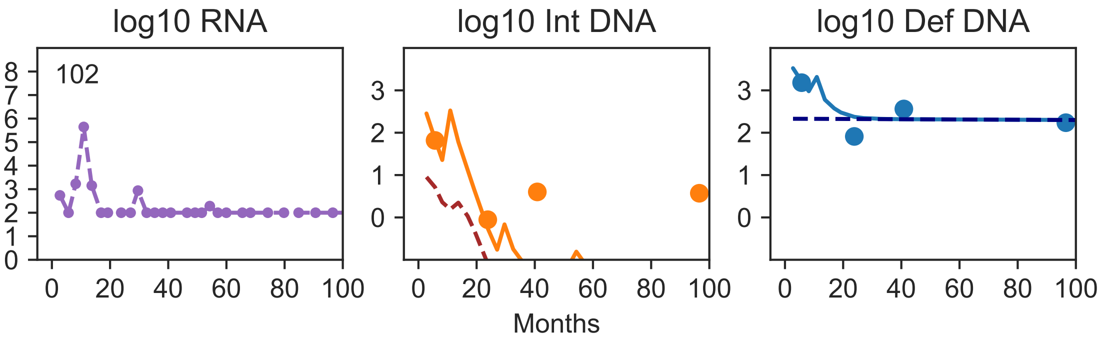

log10 RNA
log10 Int DNA
log10 Def DNA
102
8
7
6
5
4
3
2
1
0
0
20
40
60
80
100
Months

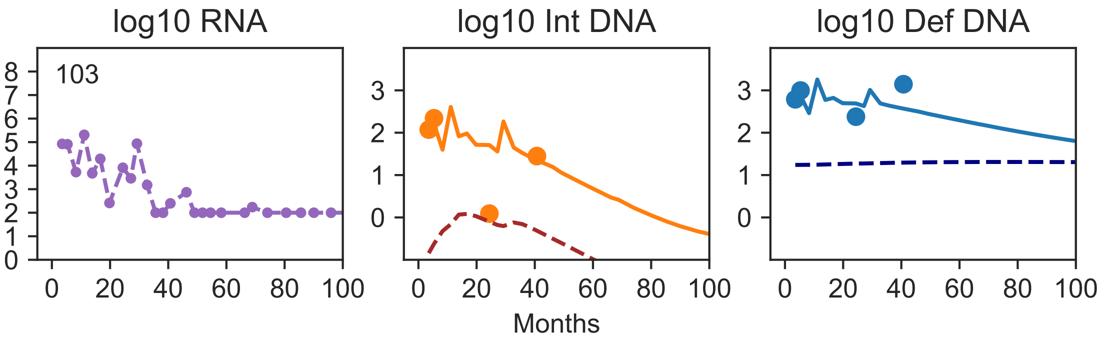

log10 RNA
log10 Int DNA
log10 Def DNA
103
Months

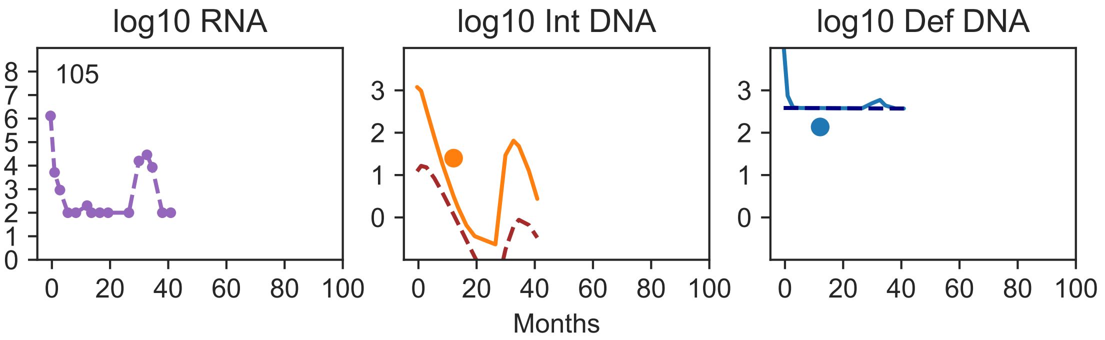

log10 RNA
log10 Int DNA
log10 Def DNA
105
Months

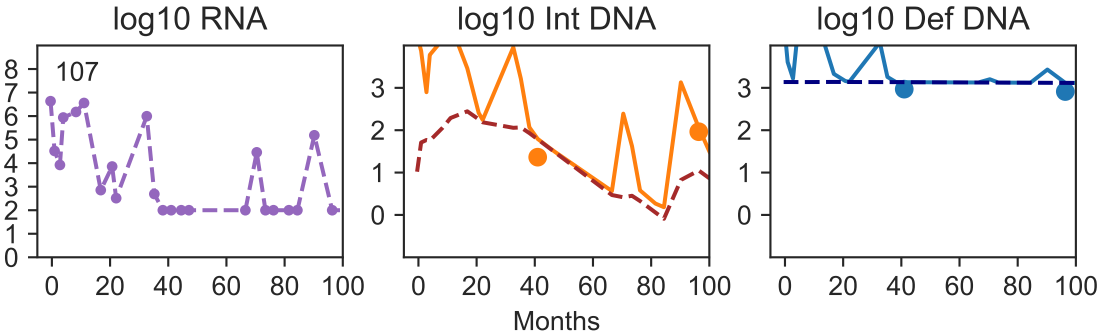

log10 RNA
107
log10 Int DNA
log10 Def DNA
Months

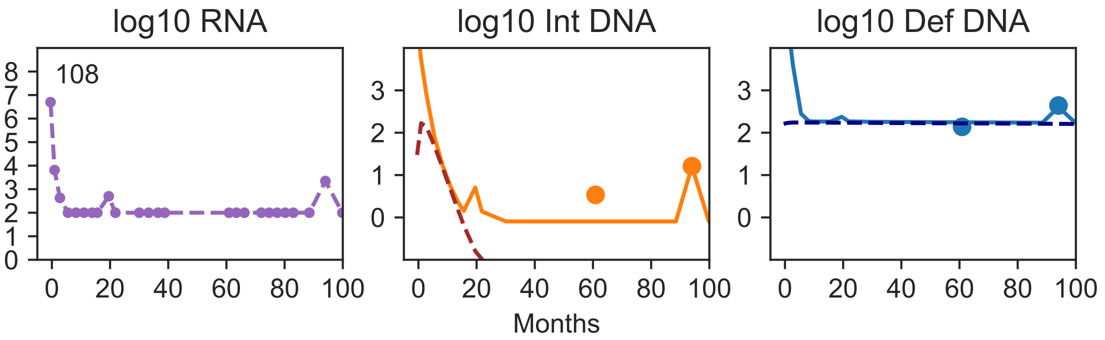

log10 RNA
log10 Int DNA
log10 Def DNA
108
0
1
2
3
4
5
6
7
8
0
20
40
60
80
100
Months

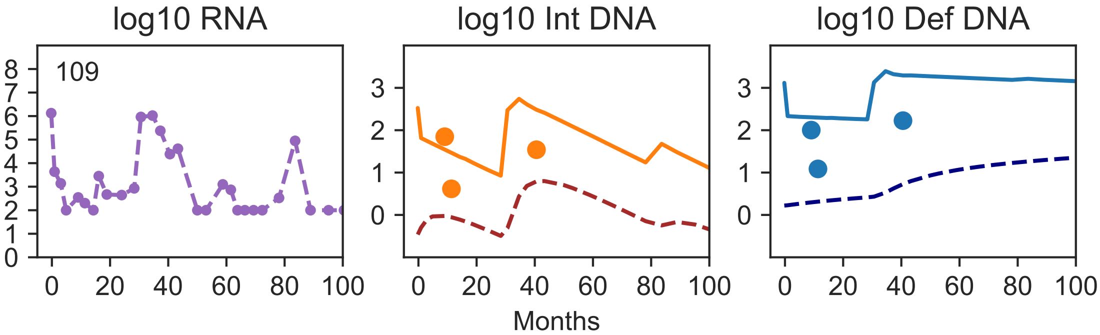

log10 RNA
log10 Int DNA
log10 Def DNA
109
0
1
2
3
4
5
6
7
8
0
1
2
3
0
1
2
3
0
20
40
60
80
100
Months

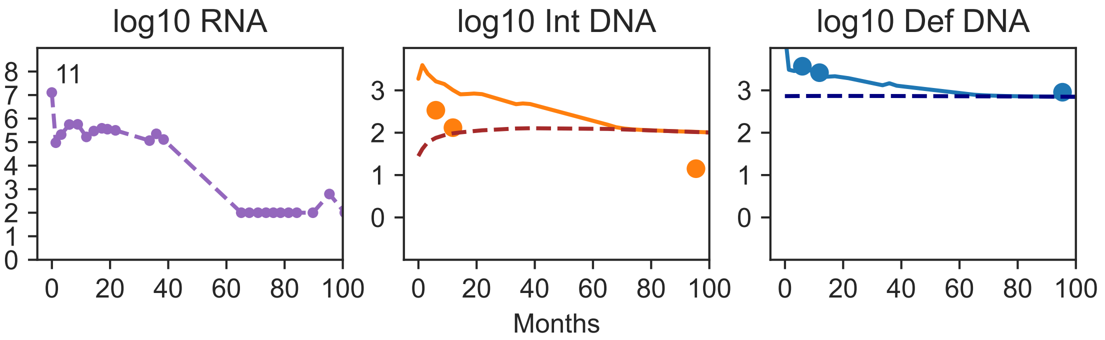

log10 RNA
log10 Int DNA
log10 Def DNA
11
0
1
2
3
4
5
6
7
8
0
20
40
60
80
100
Months

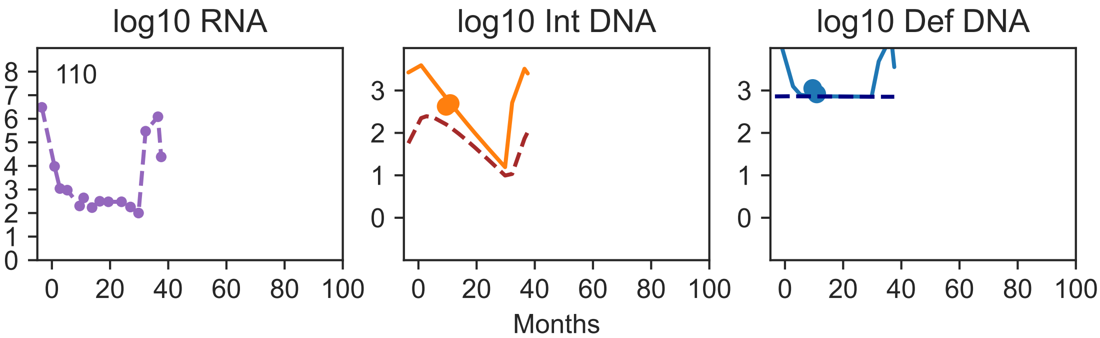
log10 RNA
log10 Int DNA
log10 Def DNA
110
0
1
2
3
4
5
6
7
8
0
20
40
60
80
100
Months

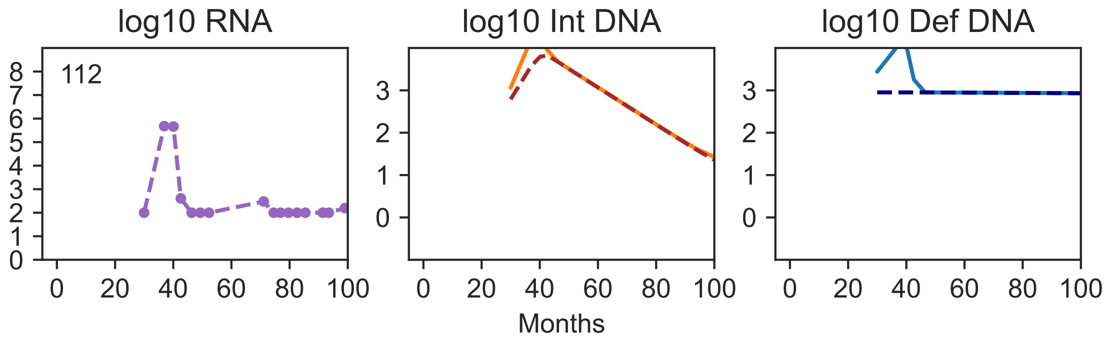

log10 RNA
log10 Int DNA
log10 Def DNA
112
Months

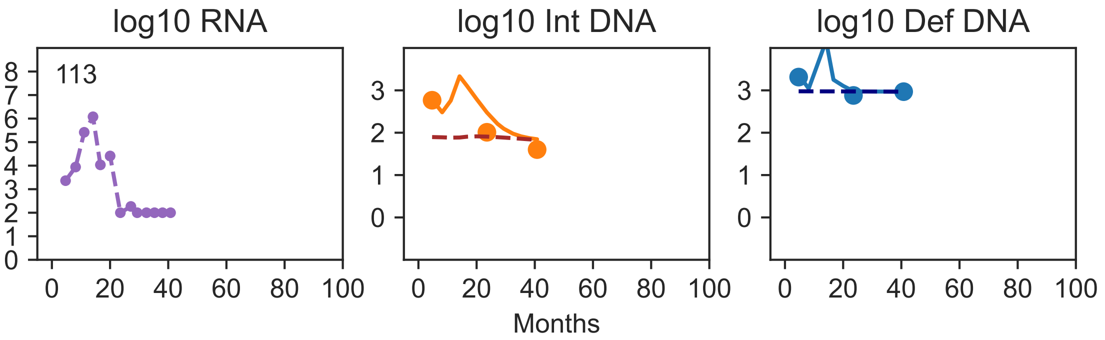
log10 RNA
log10 Int DNA
log10 Def DNA
113
Months
0 20 40 60 80 100
0 1 2 3 4 5 6 7 8
0 1 2 3

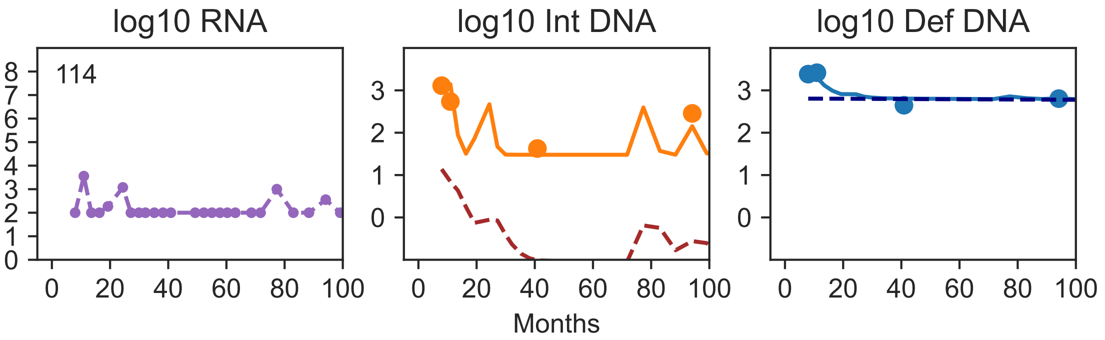

log10 RNA
log10 Int DNA
log10 Def DNA
114
Months

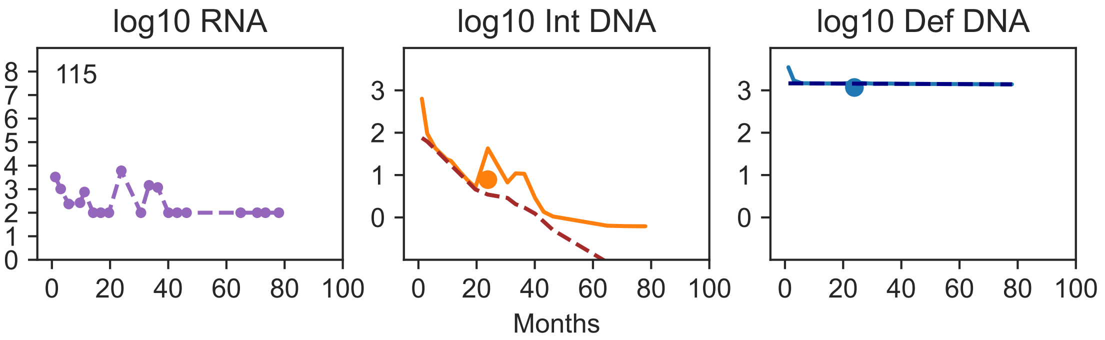

log10 RNA
115
log10 Int DNA
log10 Def DNA
Months

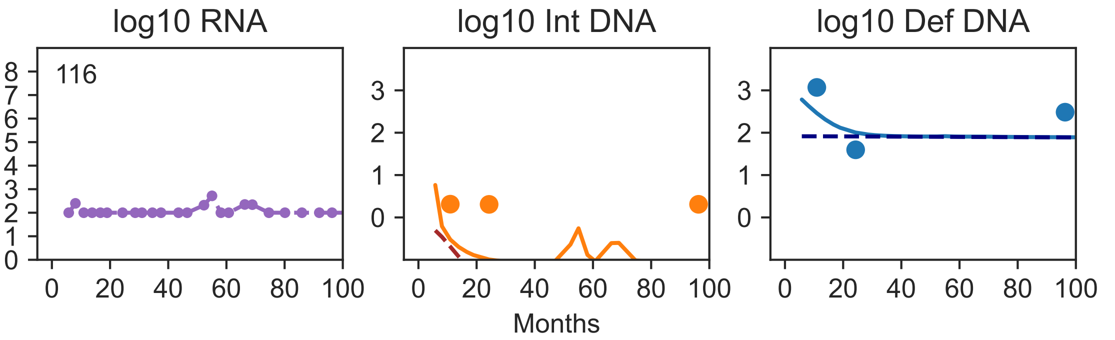

log10 RNA
log10 Int DNA
log10 Def DNA
116
Months

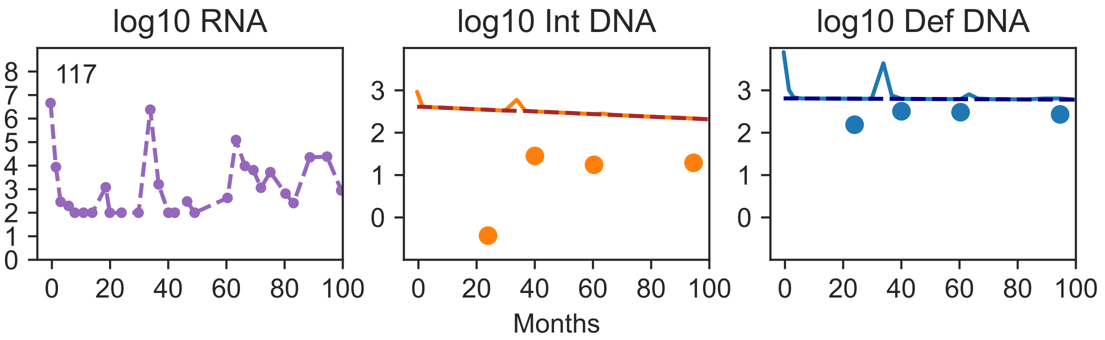

log10 RNA
log10 Int DNA
log10 Def DNA
117
0
1
2
3
4
5
6
7
8
0
20
40
60
80
100
Months

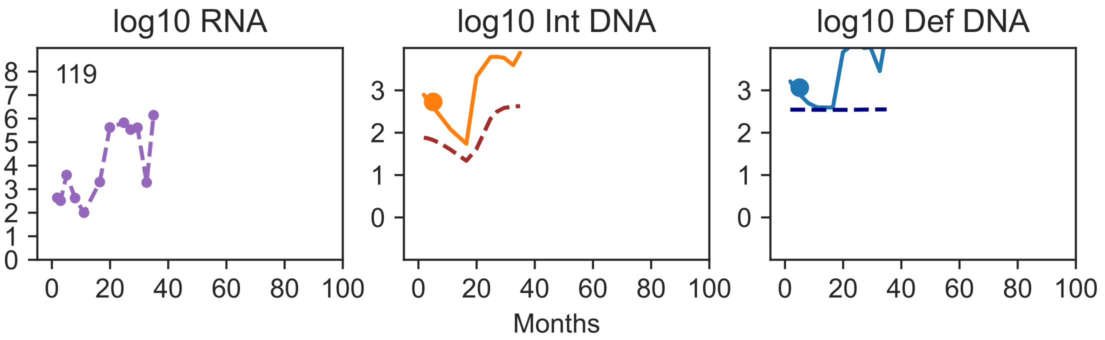

log10 RNA
log10 Int DNA
log10 Def DNA
119
0
1
2
3
4
5
6
7
8
0
20
40
60
80
100
Months

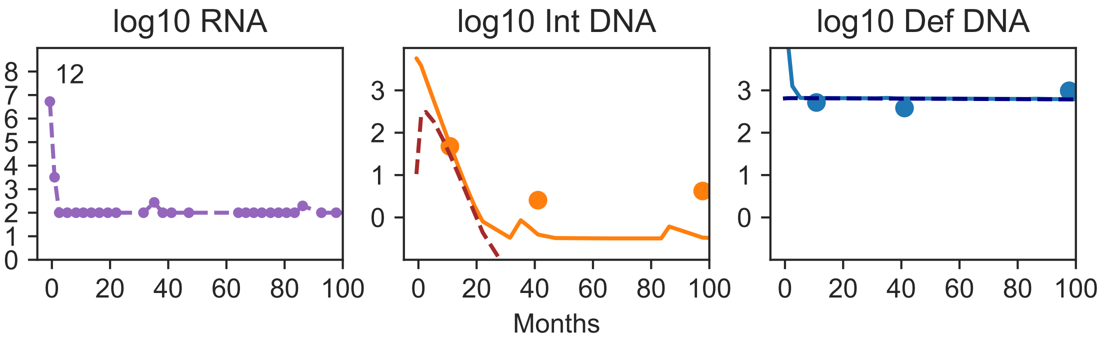

log10 RNA
log10 Int DNA
log10 Def DNA
12
0
1
2
3
4
5
6
7
8
0
20
40
60
80
100
Months

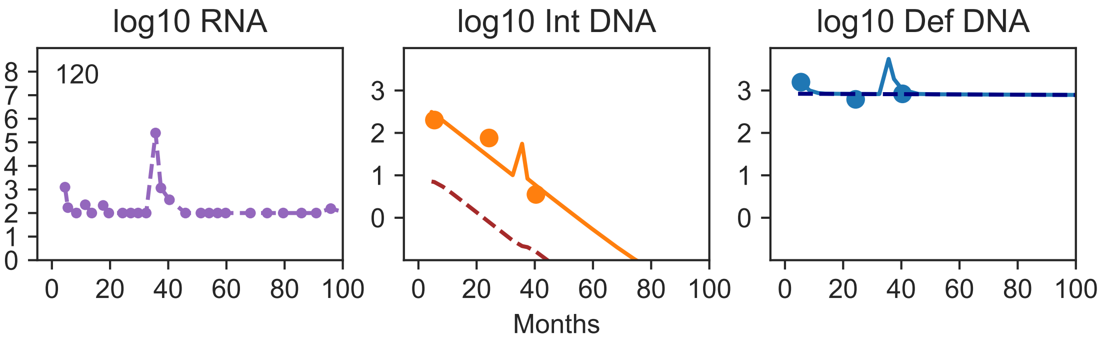
log10 RNA
120
0
1
2
3
4
5
6
7
8
0
20
40
60
80
100
log10 Int DNA
0
1
2
3
0
20
40
60
80
100
Months
log10 Def DNA
0
1
2
3
0
20
40
60
80
100

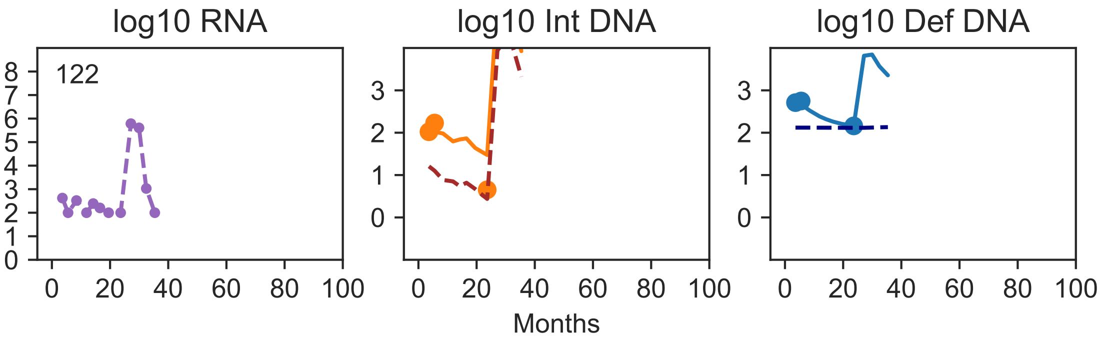

log10 RNA
log10 Int DNA
log10 Def DNA
122
0
1
2
3
4
5
6
7
8
0
20
40
60
80
100
Months

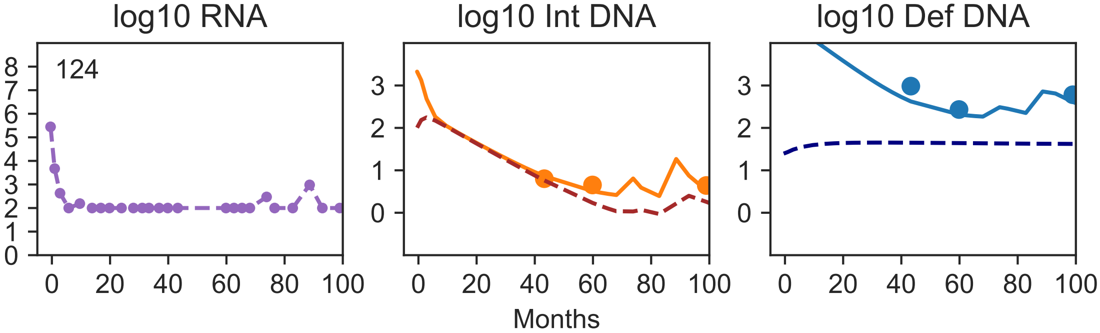

log10 RNA
log10 Int DNA
log10 Def DNA
124
0
1
2
3
4
5
6
7
8
0
20
40
60
80
100
Months

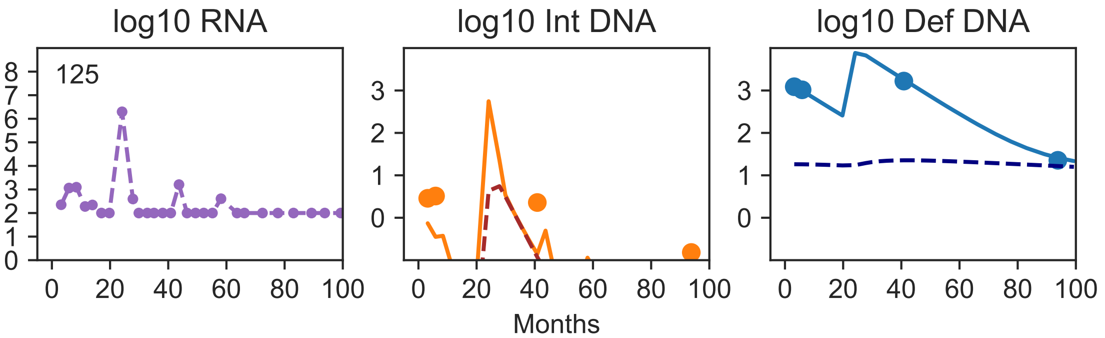

log10 RNA
125
8
7
6
5
4
3
2
1
0
0
20
40
60
80
100
log10 Int DNA
3
2
1
0
0
20
40
60
80
100
Months
log10 Def DNA
3
2
1
0
0
20
40
60
80
100

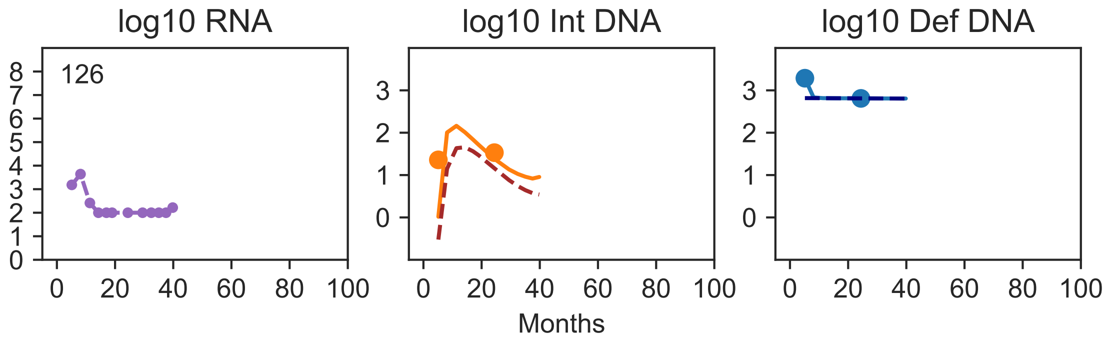

log10 RNA
log10 Int DNA
log10 Def DNA
126
Months

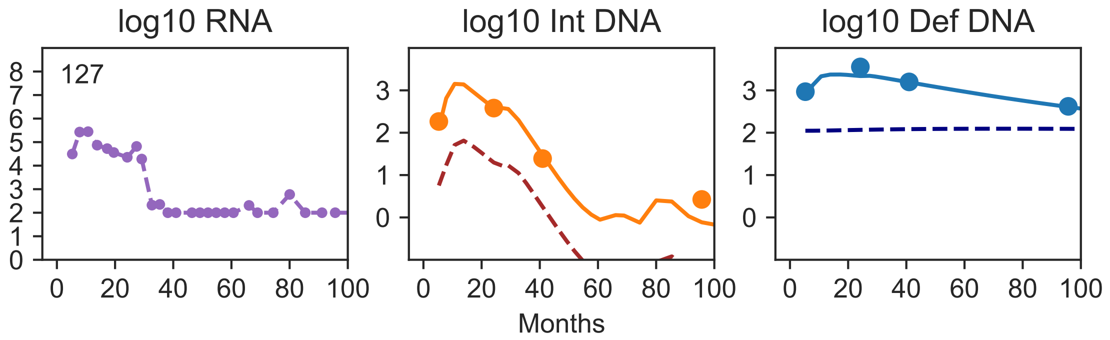

log10 RNA
127
log10 Int DNA
log10 Def DNA
Months

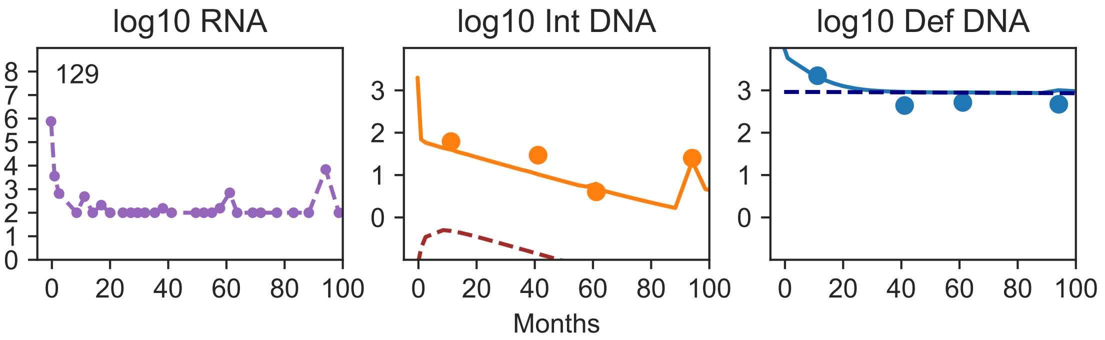

log10 RNA
log10 Int DNA
log10 Def DNA
129
Months

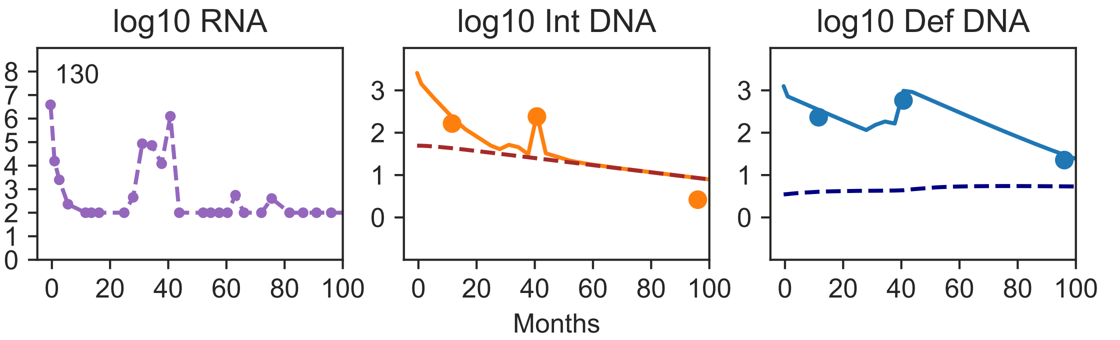

log10 RNA
log10 Int DNA
log10 Def DNA
130
8
7
6
5
4
3
2
1
0
0
20
40
60
80
100
Months

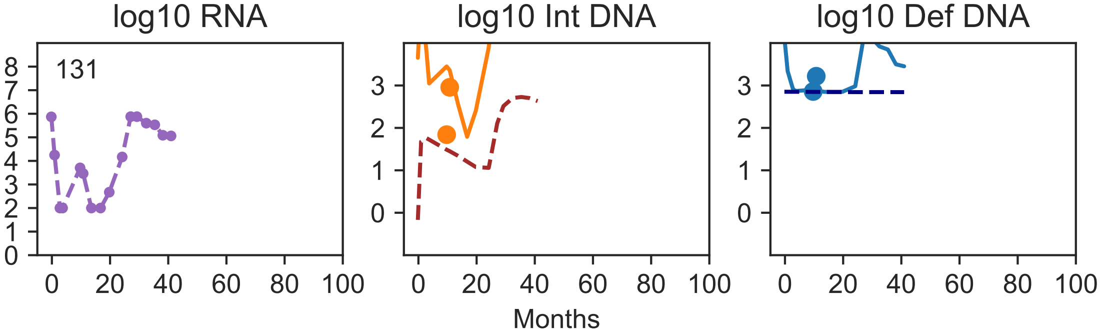

log10 RNA
log10 Int DNA
log10 Def DNA
131
0
1
2
3
4
5
6
7
8
0
20
40
60
80
100
Months

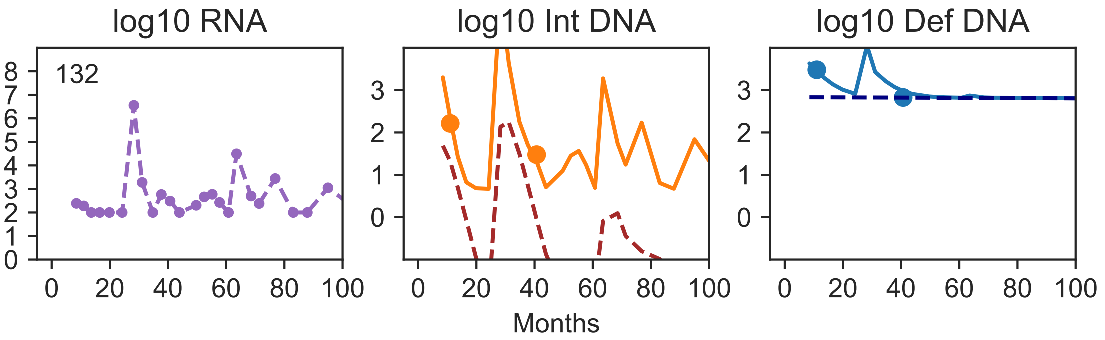

log10 RNA
log10 Int DNA
log10 Def DNA
132
0
1
2
3
4
5
6
7
8
0
20
40
60
80
100
Months

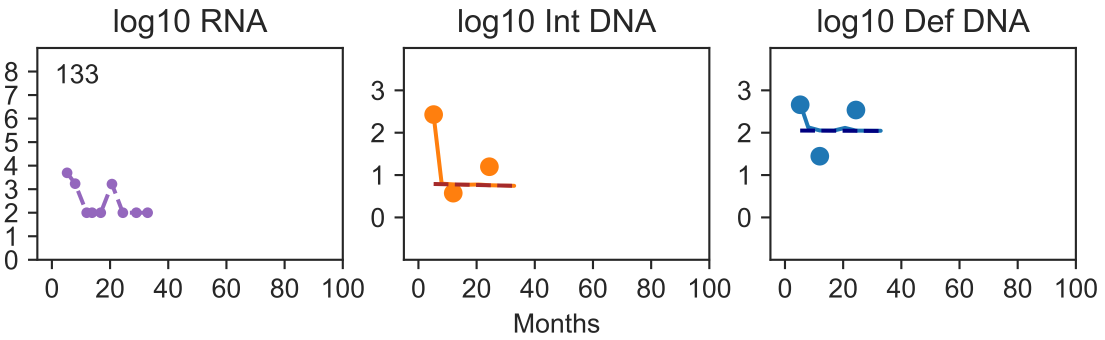
log10 RNA
log10 Int DNA
log10 Def DNA
133
Months

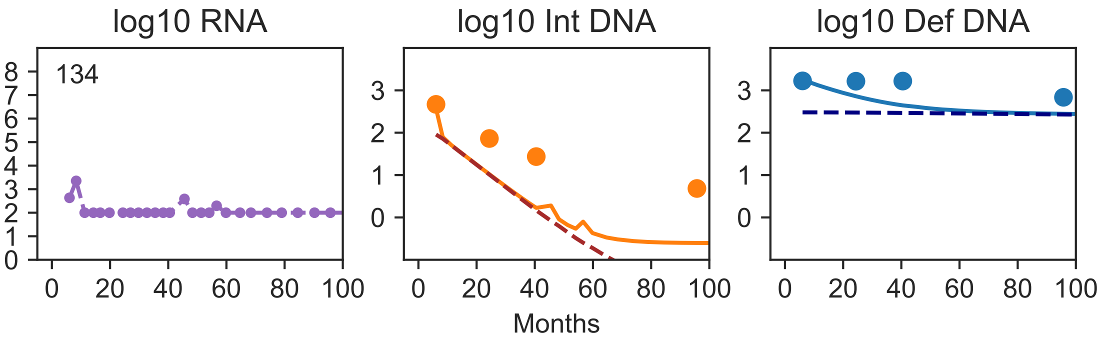

log10 RNA
log10 Int DNA
log10 Def DNA
134
Months

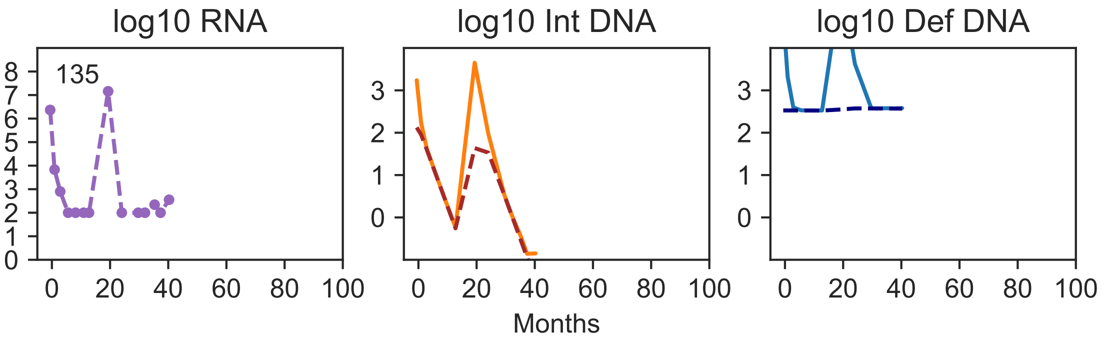
log10 RNA
log10 Int DNA
log10 Def DNA
135
0
1
2
3
4
5
6
7
8
0
20
40
60
80
100
Months

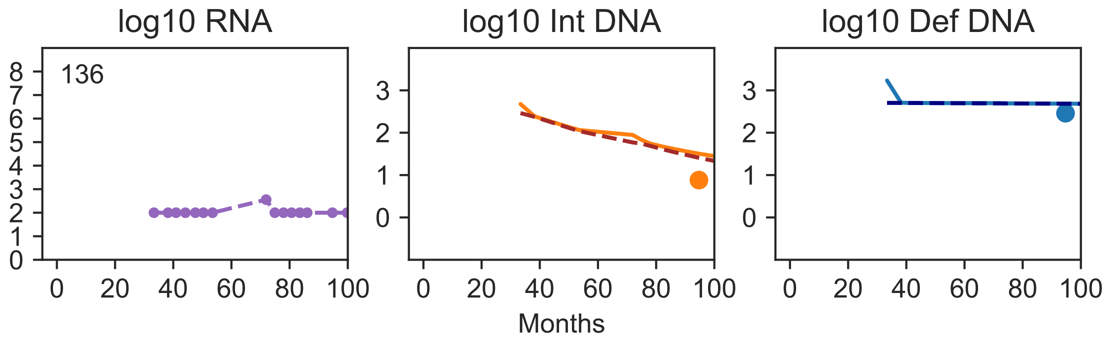

log10 RNA
log10 Int DNA
log10 Def DNA
136
Months

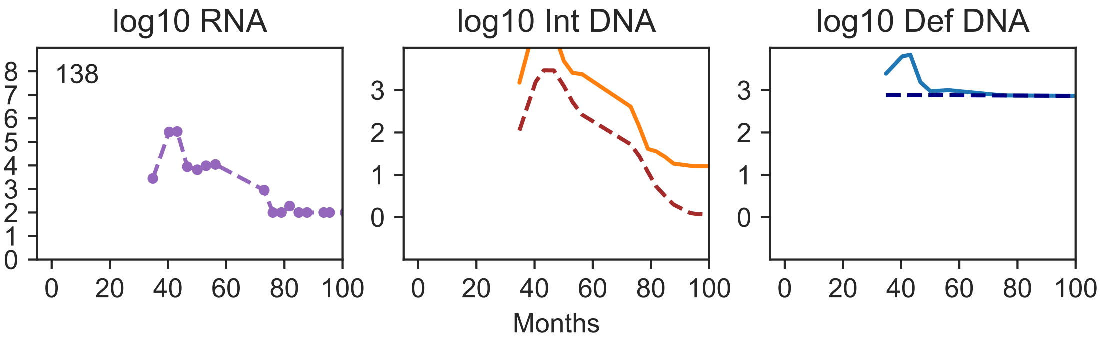

log10 RNA
log10 Int DNA
log10 Def DNA
138
Months

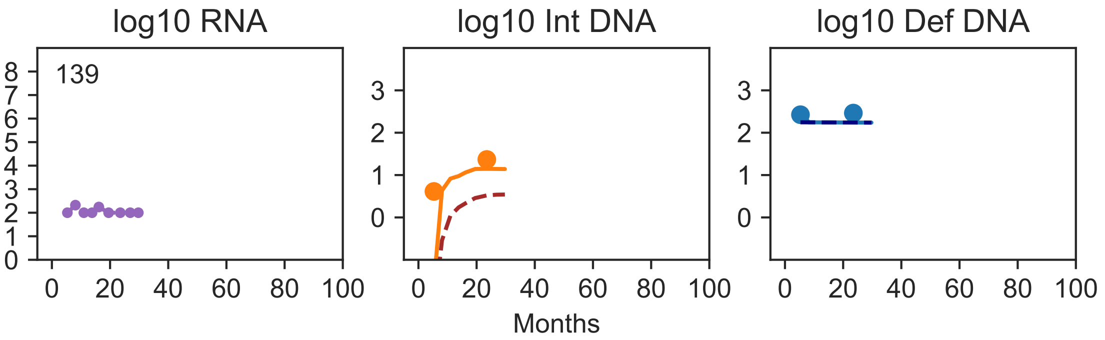

log10 RNA
log10 Int DNA
log10 Def DNA
139
8
7
6
5
4
3
2
1
0
3
2
1
0
3
2
1
0
0
20
40
60
80
100
Months

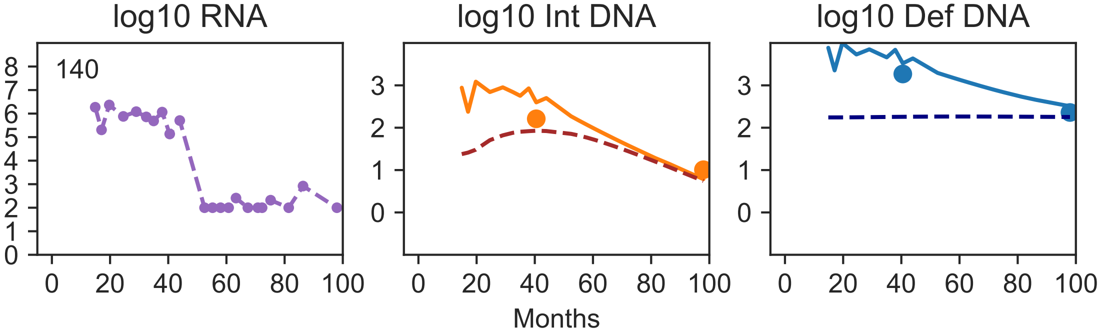
log10 RNA
log10 Int DNA
log10 Def DNA
140
Months
8
7
6
5
4
3
2
1
0
0
20
40
60
80
100

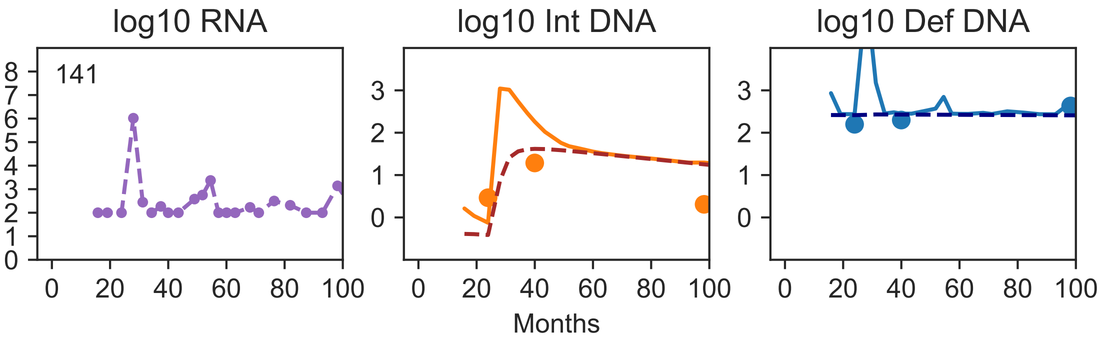
log10 RNA
log10 Int DNA
log10 Def DNA
141
Months

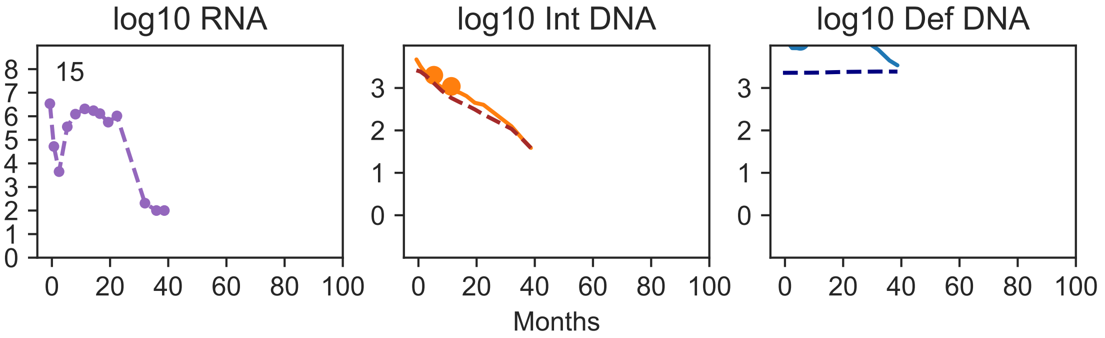

log10 RNA
log10 Int DNA
log10 Def DNA
15
8
7
6
5
4
3
2
1
0
0
20
40
60
80
100
Months

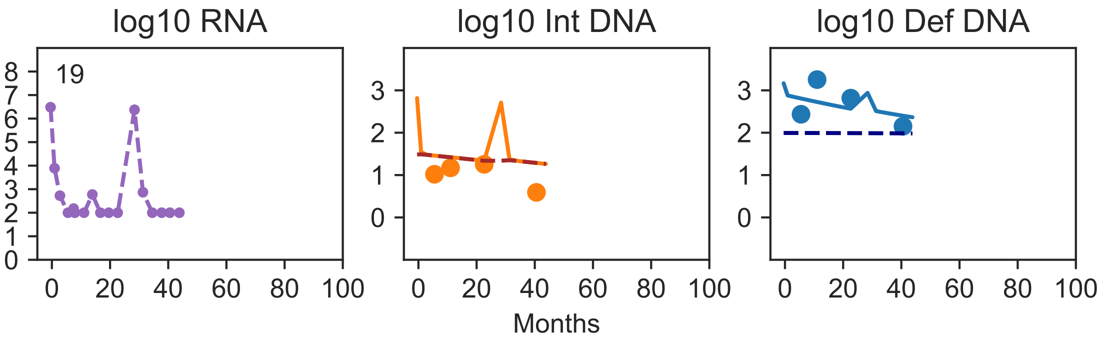

log10 RNA
log10 Int DNA
log10 Def DNA
19
0
1
2
3
4
5
6
7
8
0
20
40
60
80
100
Months

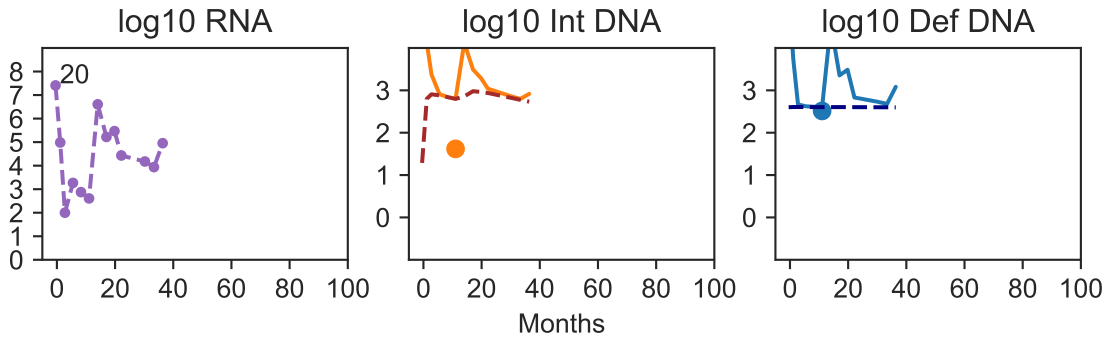

log10 RNA
log10 Int DNA
log10 Def DNA
20
0
1
2
3
4
5
6
7
8
0
20
40
60
80
100
Months

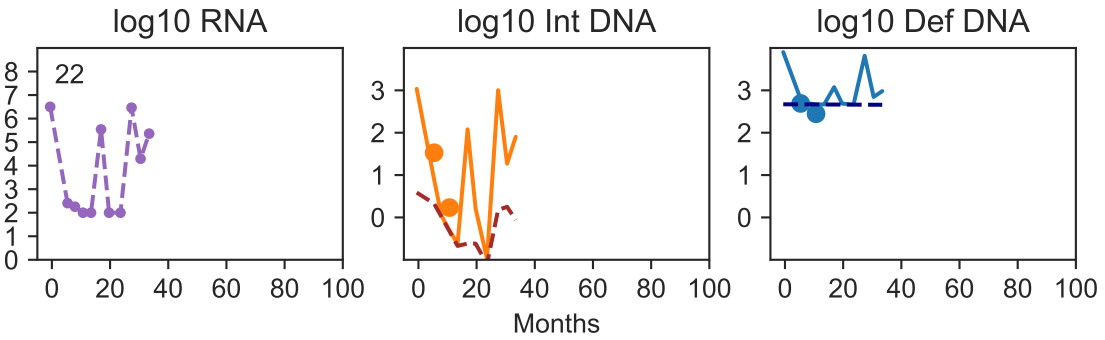
log10 RNA
log10 Int DNA
log10 Def DNA
22
Months
0
1
2
3
4
5
6
7
8
0
20
40
60
80
100

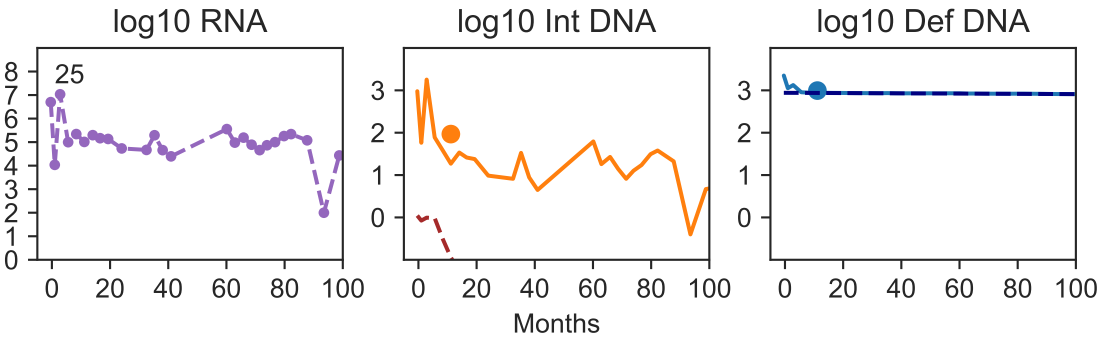

log10 RNA
log10 Int DNA
log10 Def DNA
25
0
1
2
3
4
5
6
7
8
0
20
40
60
80
100
Months

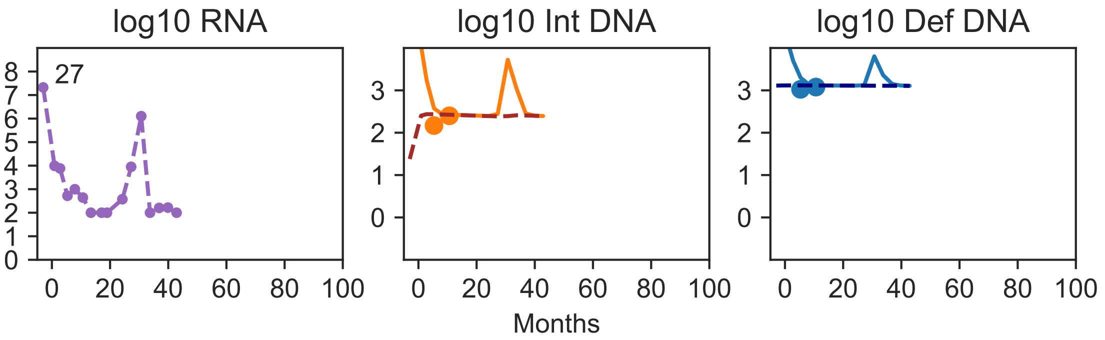

log10 RNA
log10 Int DNA
log10 Def DNA
27
Months

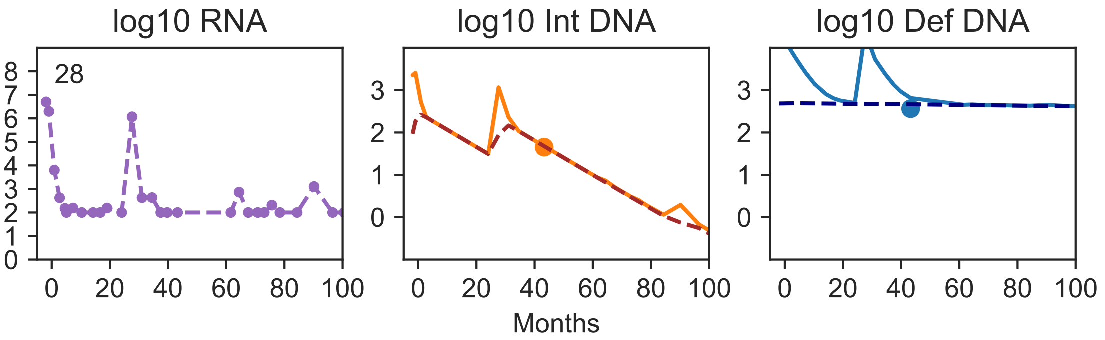

log10 RNA
28
log10 Int DNA
log10 Def DNA
Months

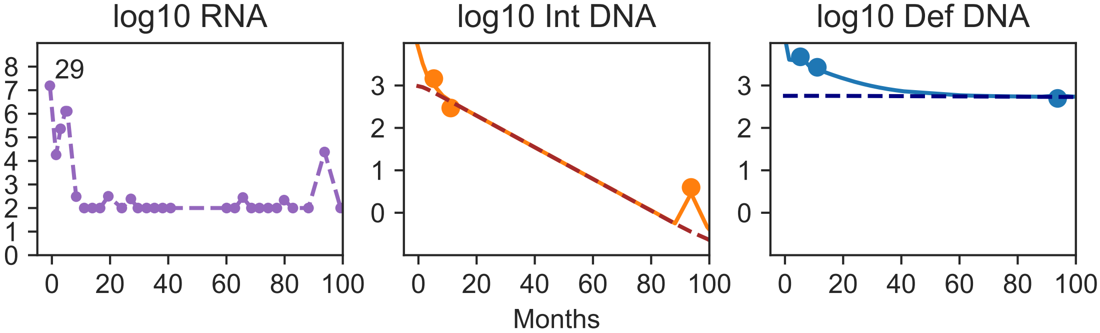
log10 RNA
log10 Int DNA
log10 Def DNA
29
Months
0
1
2
3
4
5
6
7
8
0
20
40
60
80
100

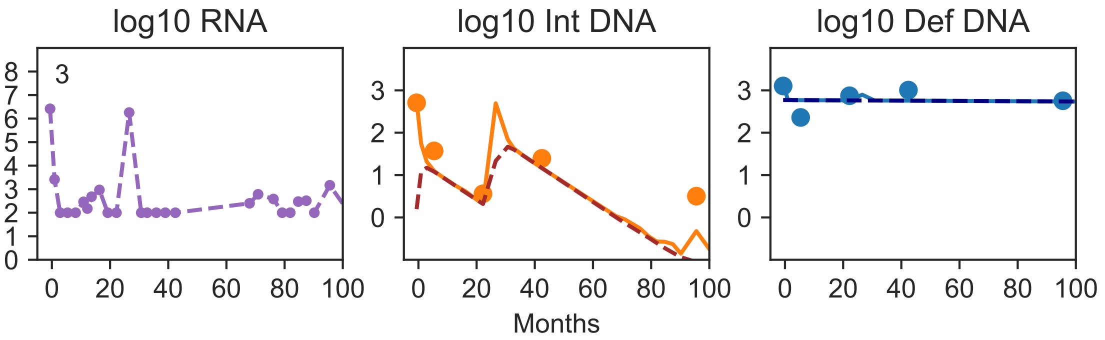
log10 RNA
log10 Int DNA
log10 Def DNA
3
Months

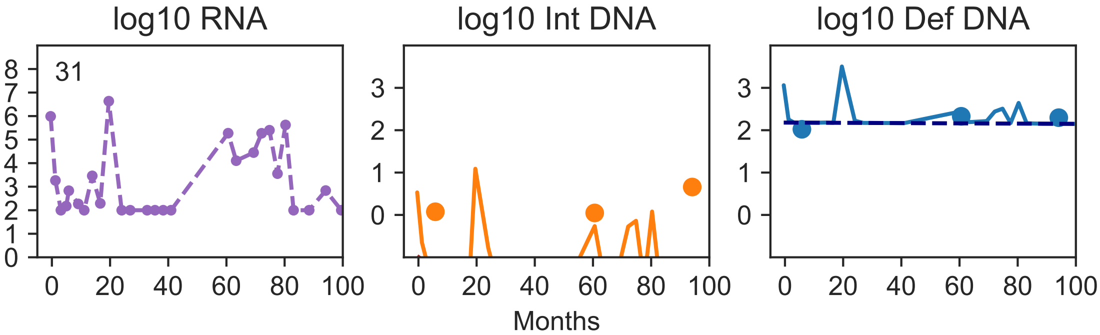

log10 RNA
log10 Int DNA
log10 Def DNA
31
0
1
2
3
4
5
6
7
8
0
20
40
60
80
100
Months

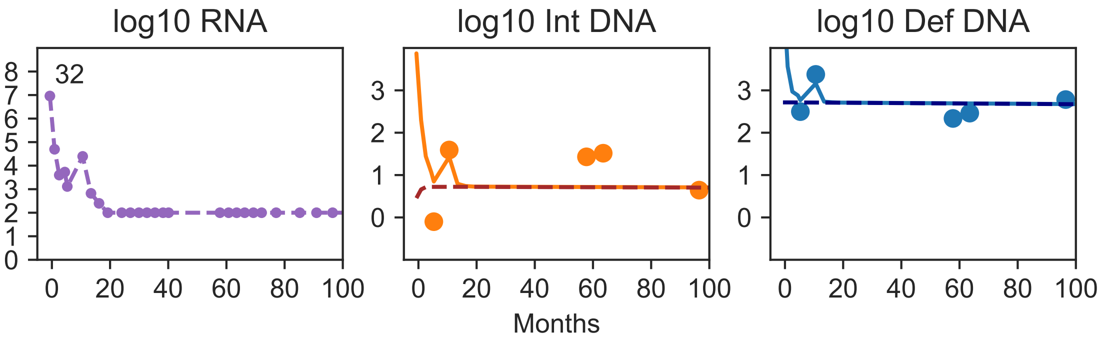

log10 RNA
log10 Int DNA
log10 Def DNA
32
0
1
2
3
4
5
6
7
8
0
20
40
60
80
100
Months

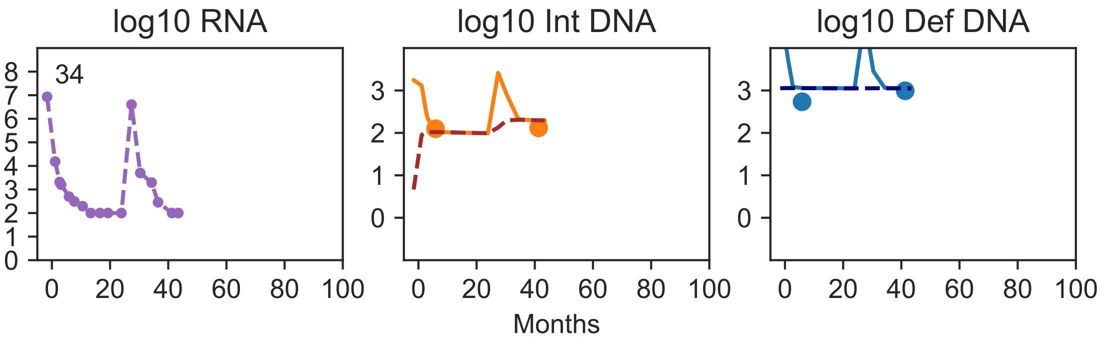

log10 RNA
log10 Int DNA
log10 Def DNA
34
0
1
2
3
4
5
6
7
8
0
1
2
3
0
20
40
60
80
100
Months

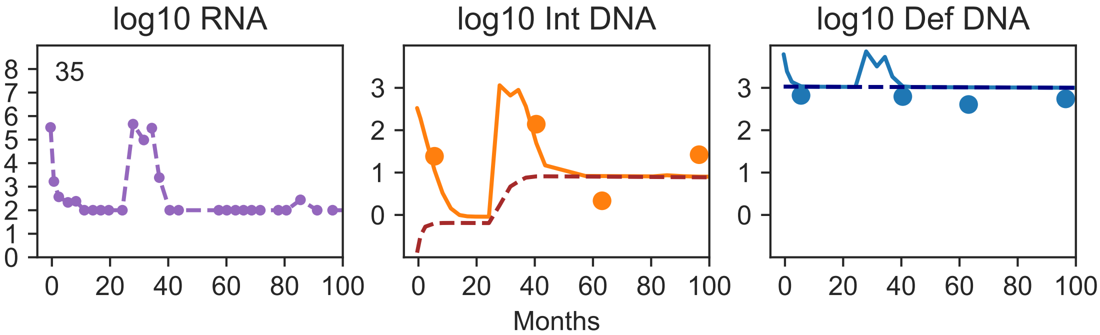

log10 RNA
log10 Int DNA
log10 Def DNA
35
Months

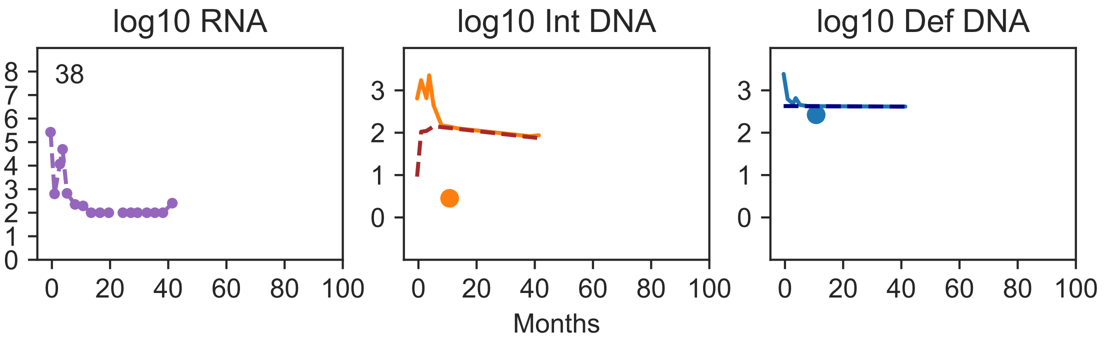

log10 RNA
38
log10 Int DNA
log10 Def DNA
Months

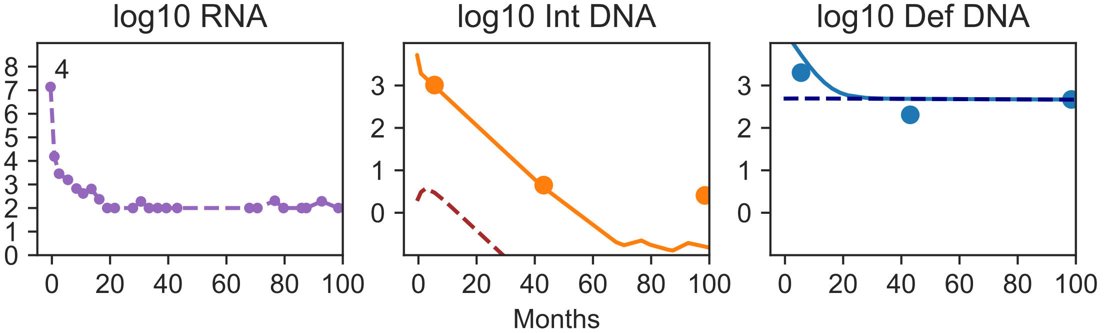

log10 RNA
log10 Int DNA
log10 Def DNA
4
0
1
2
3
4
5
6
7
8
0
20
40
60
80
100
Months

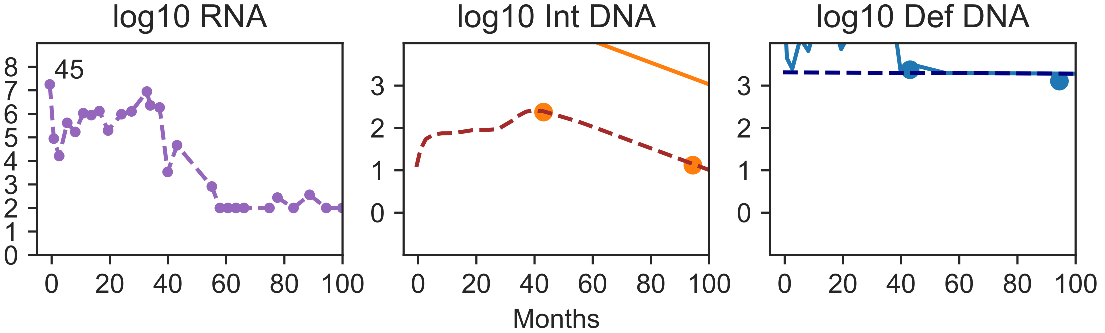

log10 RNA
log10 Int DNA
log10 Def DNA
45
0
1
2
3
4
5
6
7
8
0
20
40
60
80
100
Months

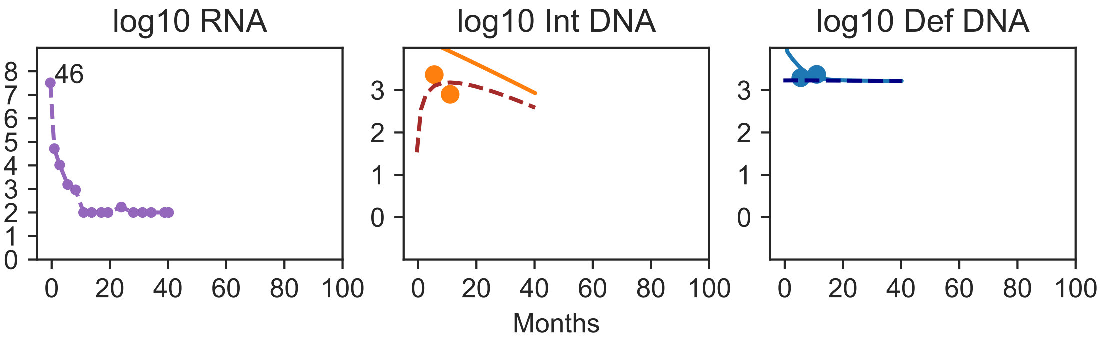

log10 RNA
log10 Int DNA
log10 Def DNA
46
0
1
2
3
4
5
6
7
8
0
20
40
60
80
100
Months

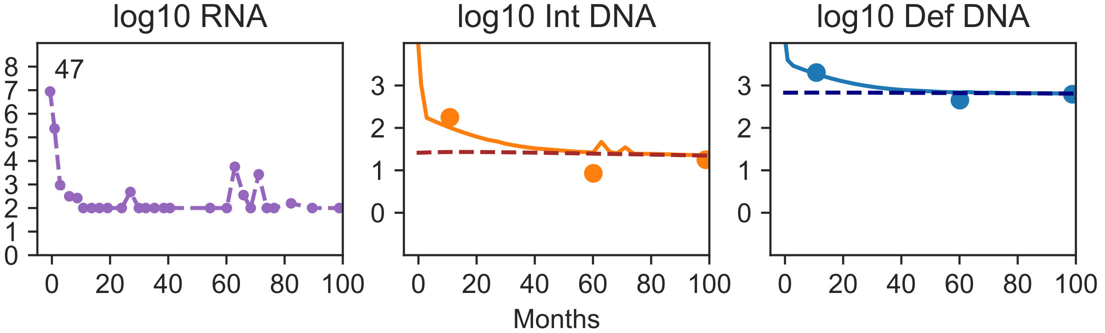

log10 RNA
log10 Int DNA
log10 Def DNA
47
8
7
6
5
4
3
2
1
0
3
2
1
0
3
2
1
0
0
20
40
60
80
100
0
20
40
60
80
100
0
20
40
60
80
100
Months

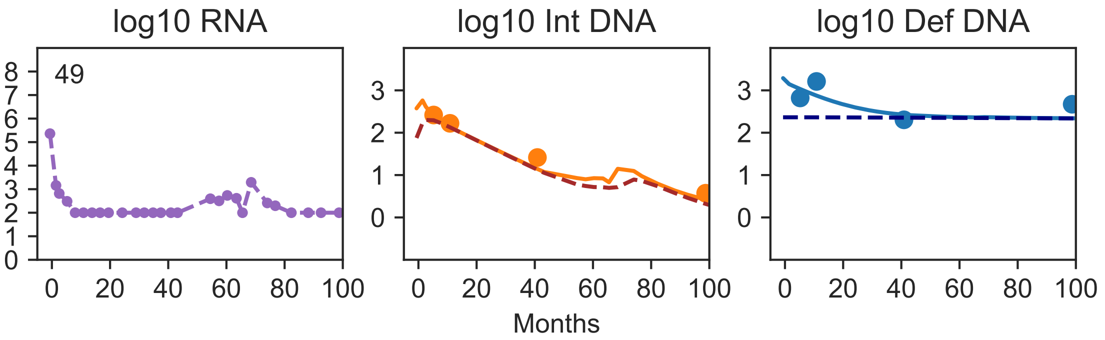

log10 RNA
log10 Int DNA
log10 Def DNA
49
0
1
2
3
4
5
6
7
8
0
20
40
60
80
100
Months

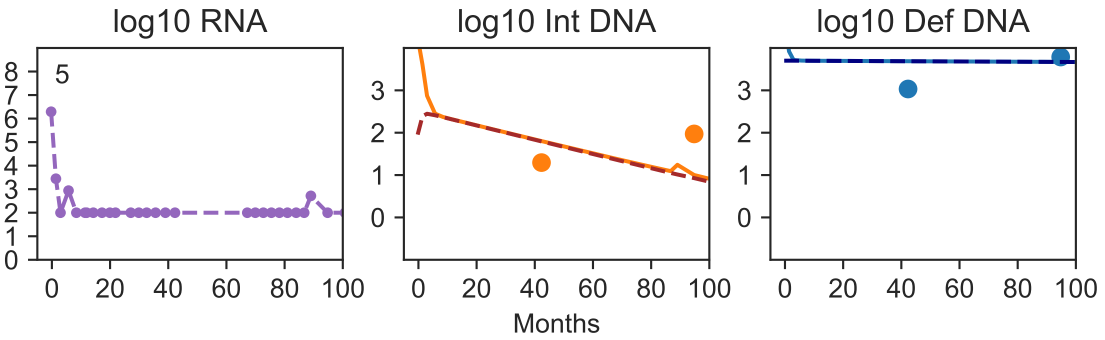

log10 RNA
log10 Int DNA
log10 Def DNA
5
0
1
2
3
4
5
6
7
8
0
20
40
60
80
100
Months

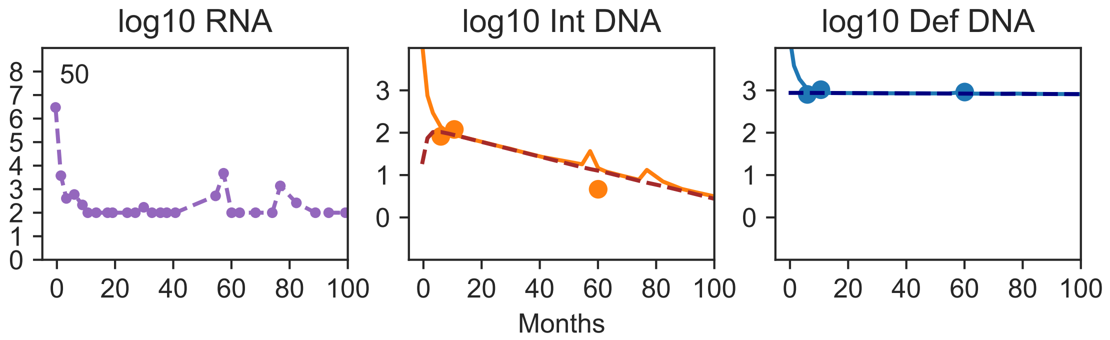

log10 RNA
log10 Int DNA
log10 Def DNA
50
8
7
6
5
4
3
2
1
0
0
20
40
60
80
100
Months

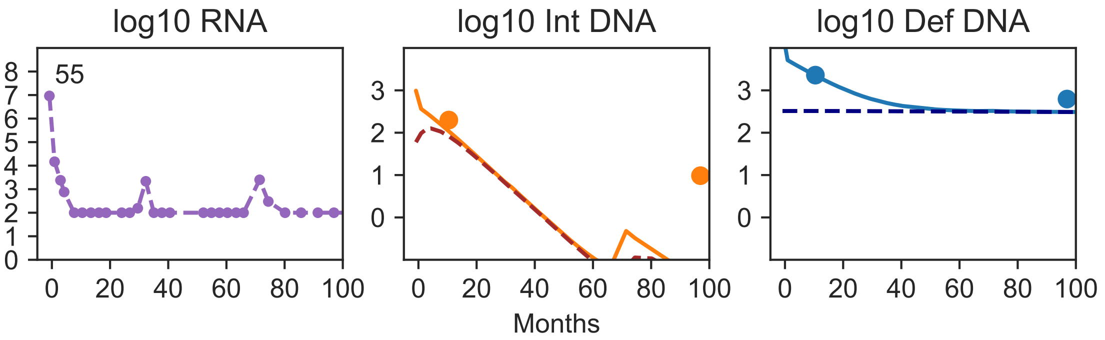

log10 RNA
log10 Int DNA
log10 Def DNA
55
Months

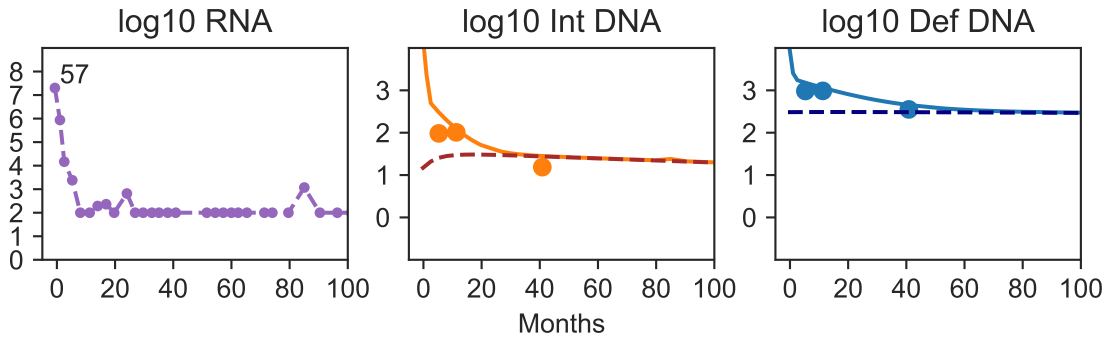

log10 RNA
log10 Int DNA
log10 Def DNA
57
8
7
6
5
4
3
2
1
0
3
2
1
0
3
2
1
0
0
20
40
60
80
100
0
20
40
60
80
100
0
20
40
60
80
100
Months

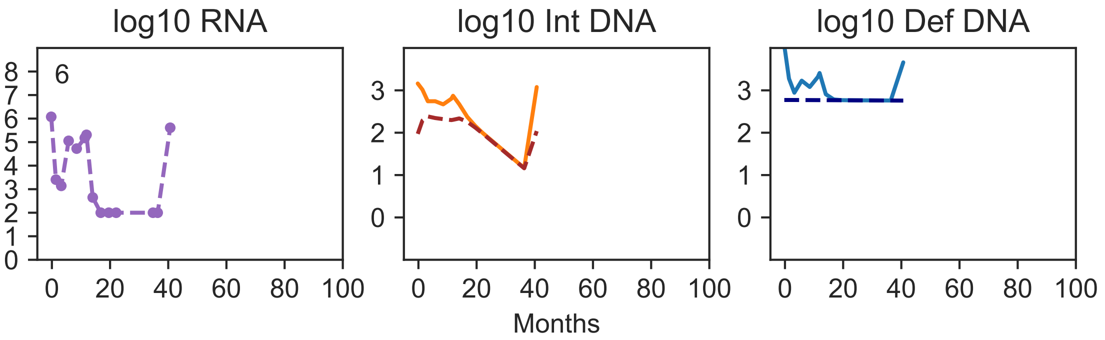

log10 RNA
log10 Int DNA
log10 Def DNA
6
Months

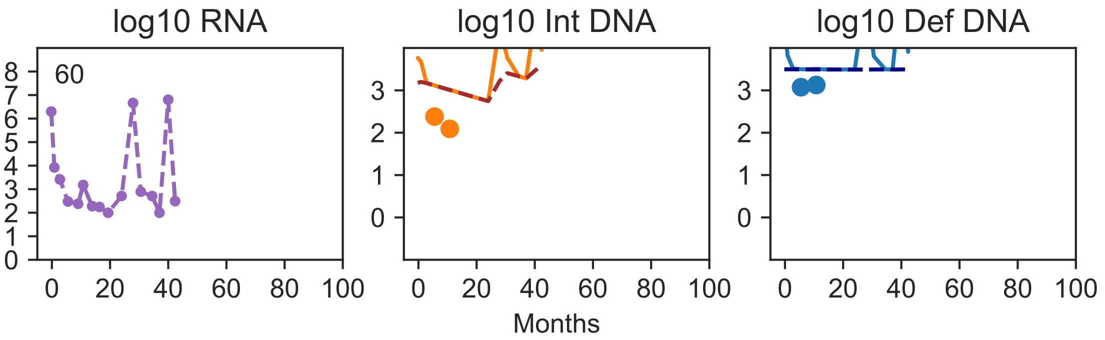

log10 RNA
60
8
7
6
5
4
3
2
1
0
0
20
40
60
80
100
log10 Int DNA
3
2
1
0
0
20
40
60
80
100
Months
log10 Def DNA
3
2
1
0
0
20
40
60
80
100

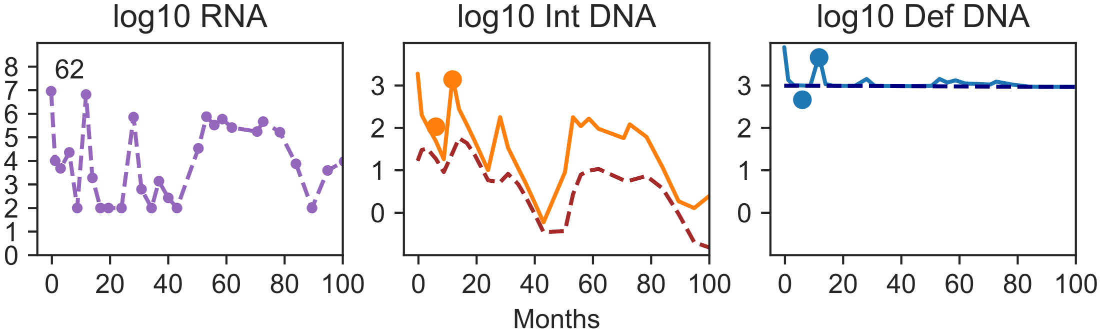

log10 RNA
log10 Int DNA
log10 Def DNA
62
0
1
2
3
4
5
6
7
8
0
20
40
60
80
100
Months

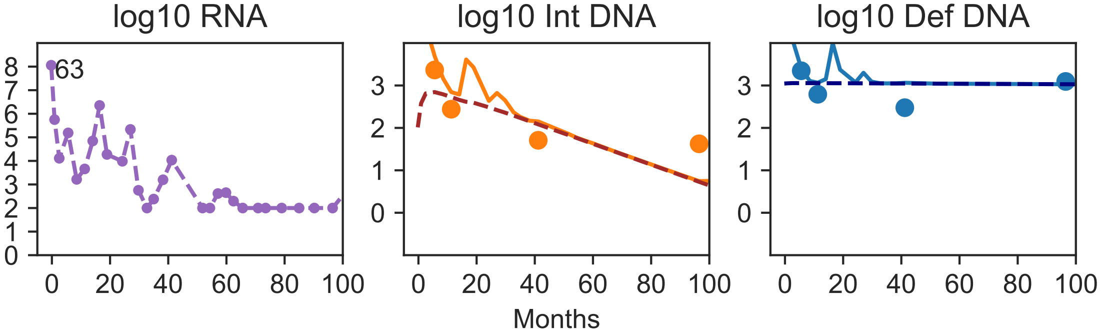

log10 RNA
log10 Int DNA
log10 Def DNA
63
Months

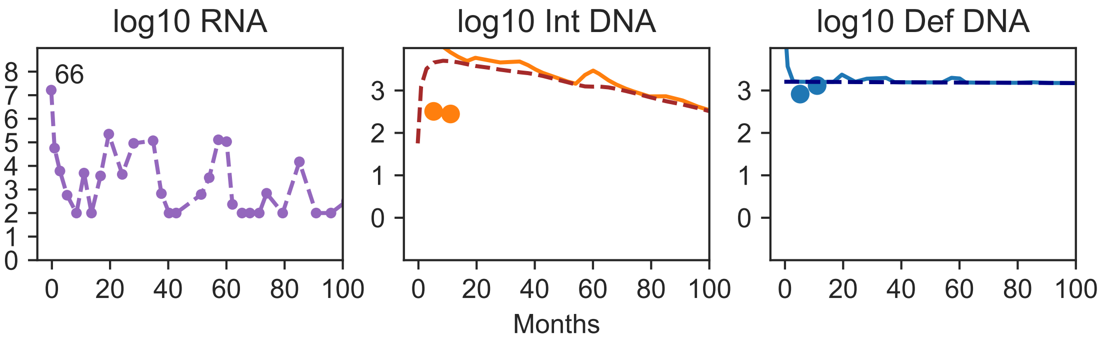

log10 RNA
log10 Int DNA
log10 Def DNA
66
Months

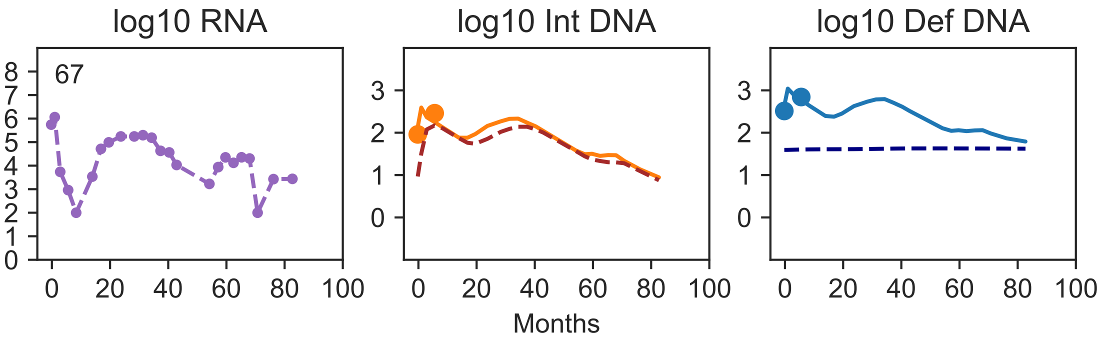
log10 RNA
log10 Int DNA
log10 Def DNA
67
0
1
2
3
4
5
6
7
8
0
20
40
60
80
100
Months

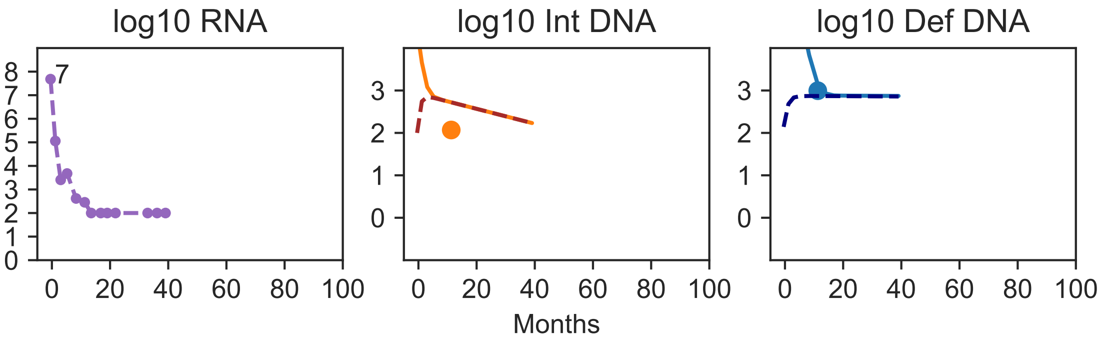

log10 RNA
log10 Int DNA
log10 Def DNA
8
7
6
5
4
3
2
1
0
7
0
20
40
60
80
100
Months

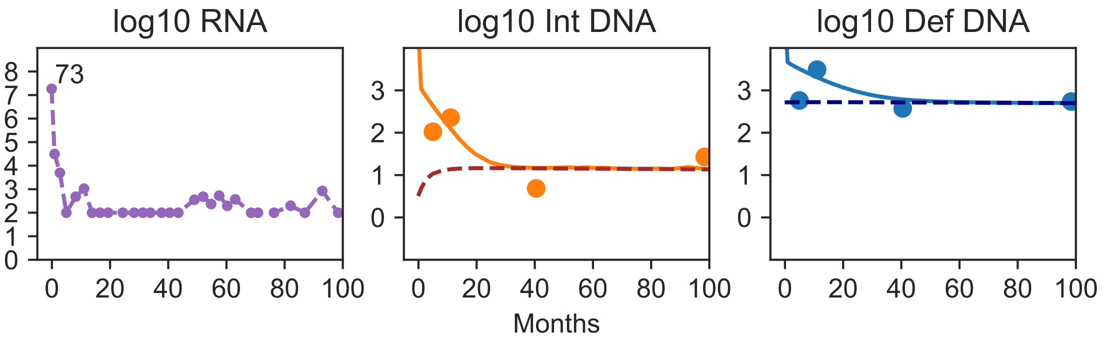
log10 RNA
log10 Int DNA
log10 Def DNA
73
0
1
2
3
4
5
6
7
8
0
20
40
60
80
100
Months

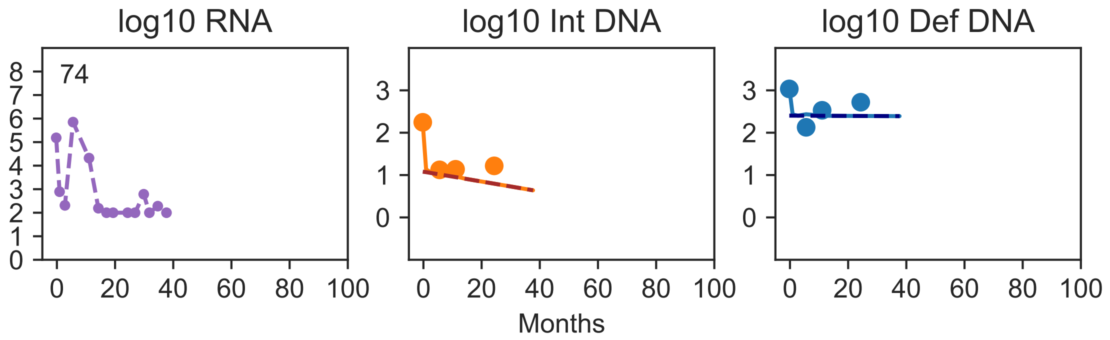

log10 RNA
log10 Int DNA
log10 Def DNA
74
Months

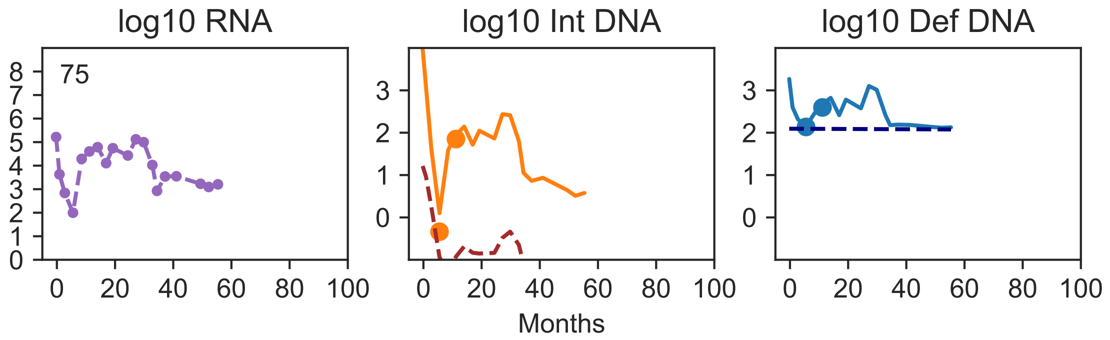
log10 RNA
log10 Int DNA
log10 Def DNA
75
Months

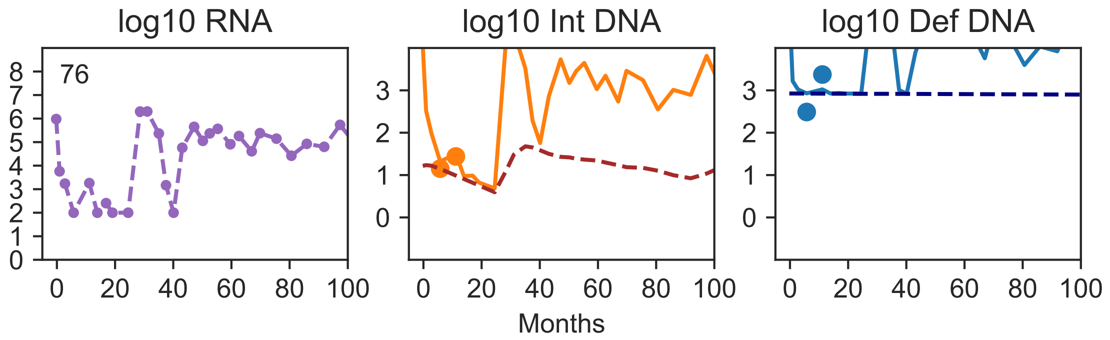

log10 RNA
log10 Int DNA
log10 Def DNA
76
0 1 2 3 4 5 6 7 8
0 1 2 3
0 20 40 60 80 100
Months

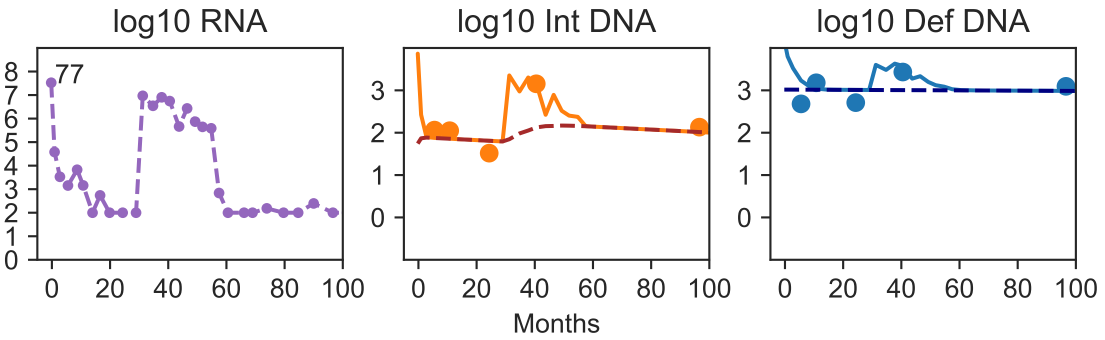

log10 RNA
log10 Int DNA
log10 Def DNA
77
0
1
2
3
4
5
6
7
8
0
20
40
60
80
100
Months

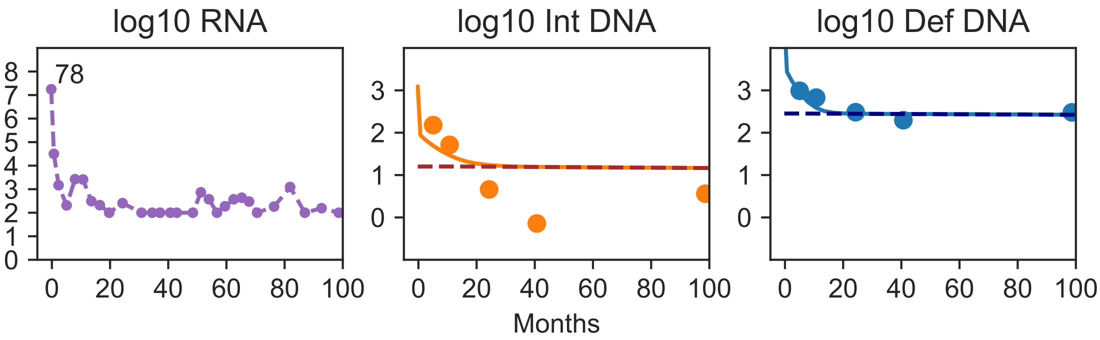

log10 RNA
log10 Int DNA
log10 Def DNA
78
8
7
6
5
4
3
2
1
0
0
20
40
60
80
100
Months

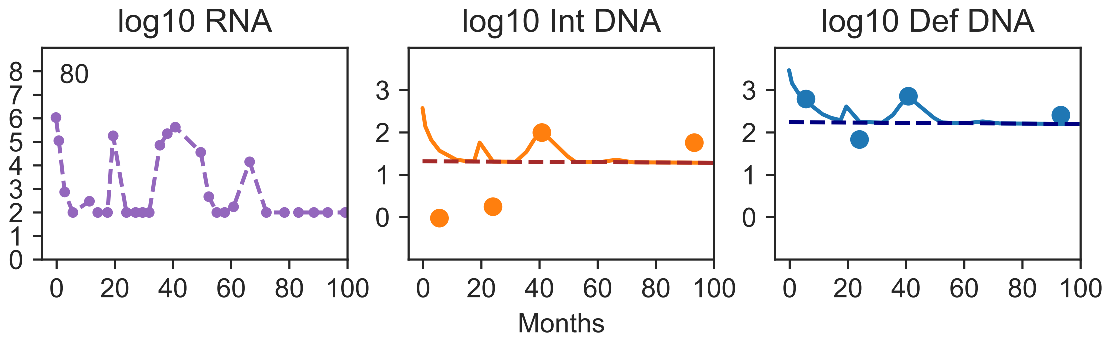

log10 RNA
log10 Int DNA
log10 Def DNA
80
Months

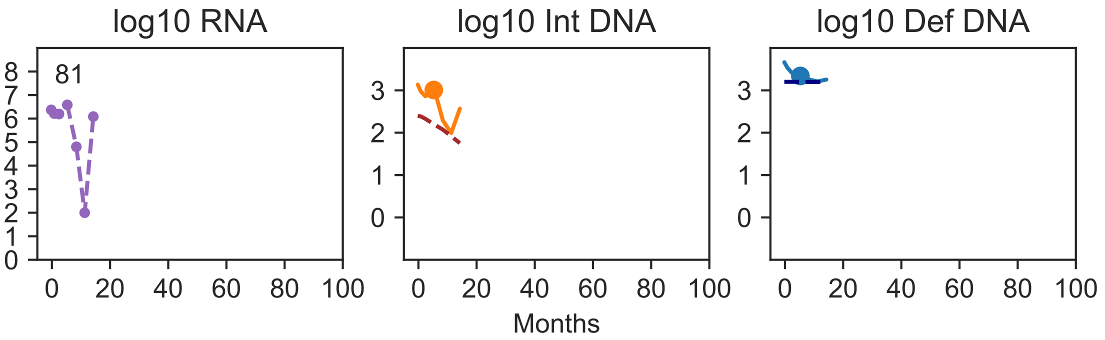

log10 RNA
log10 Int DNA
log10 Def DNA
81
0
1
2
3
4
5
6
7
8
0
20
40
60
80
100
Months

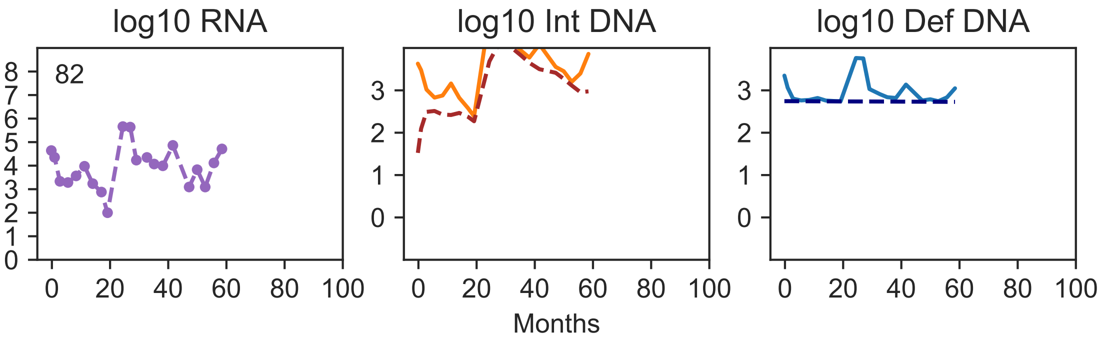

log10 RNA
log10 Int DNA
log10 Def DNA
82
8
7
6
5
4
3
2
1
0
0
20
40
60
80
100
Months

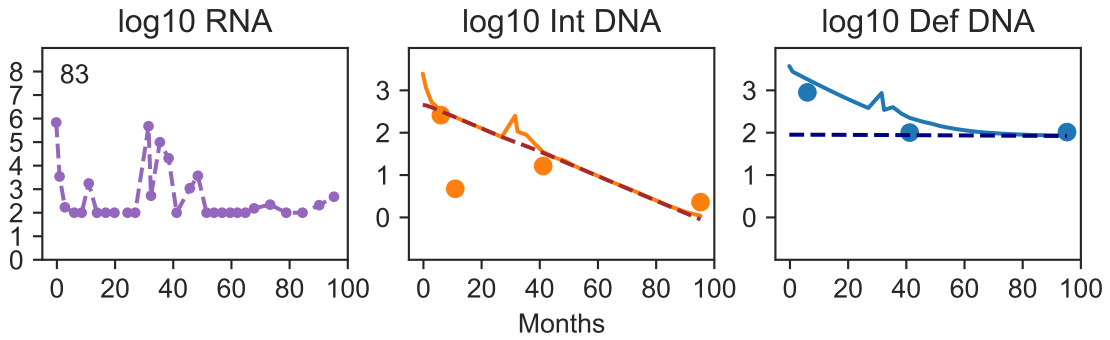
log10 RNA
log10 Int DNA
log10 Def DNA
83
0
1
2
3
4
5
6
7
8
0
20
40
60
80
100
Months

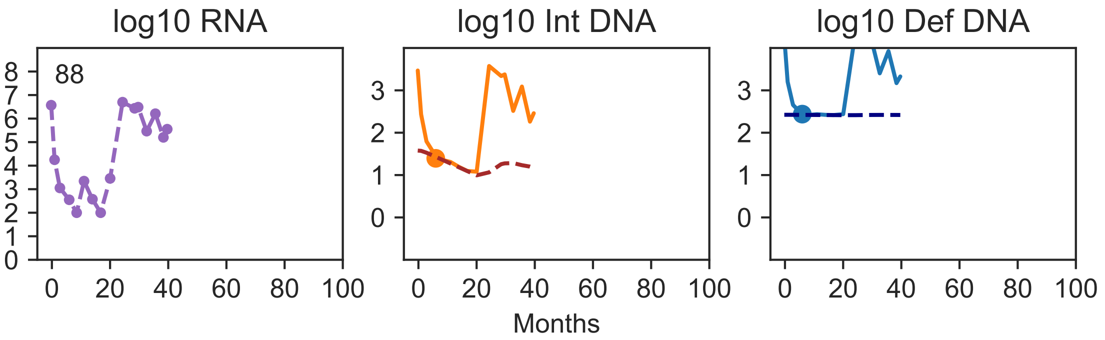

log10 RNA
log10 Int DNA
log10 Def DNA
88
0
1
2
3
4
5
6
7
8
0
20
40
60
80
100
Months

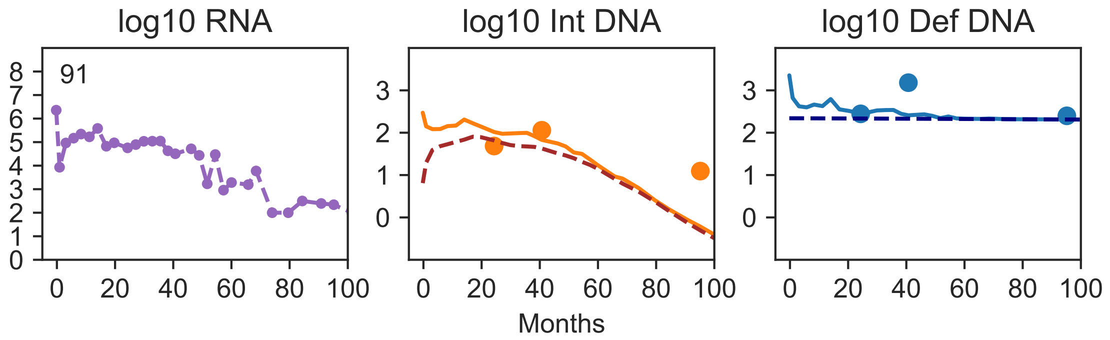

log10 RNA
log10 Int DNA
log10 Def DNA
91
Months

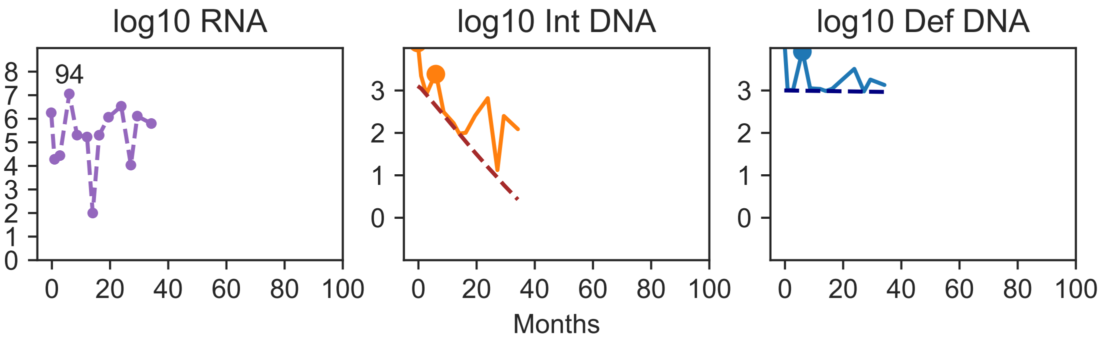

log10 RNA
94
log10 Int DNA
log10 Def DNA
Months

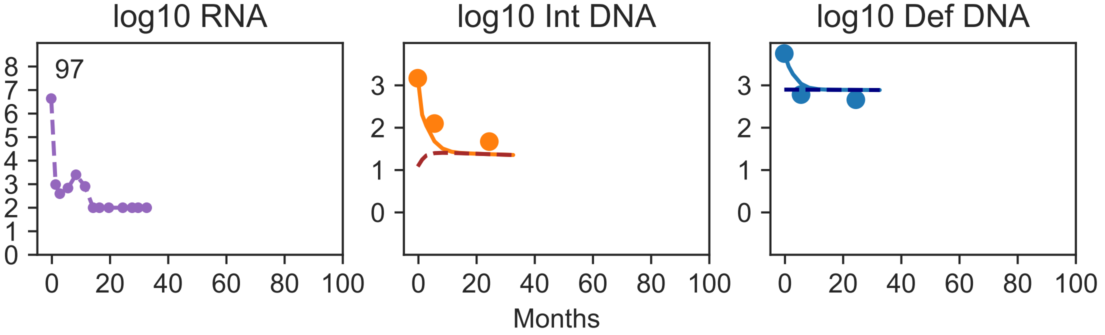

log10 RNA
log10 Int DNA
log10 Def DNA
97
0
1
2
3
4
5
6
7
8
0
1
2
3
0
20
40
60
80
100
Months

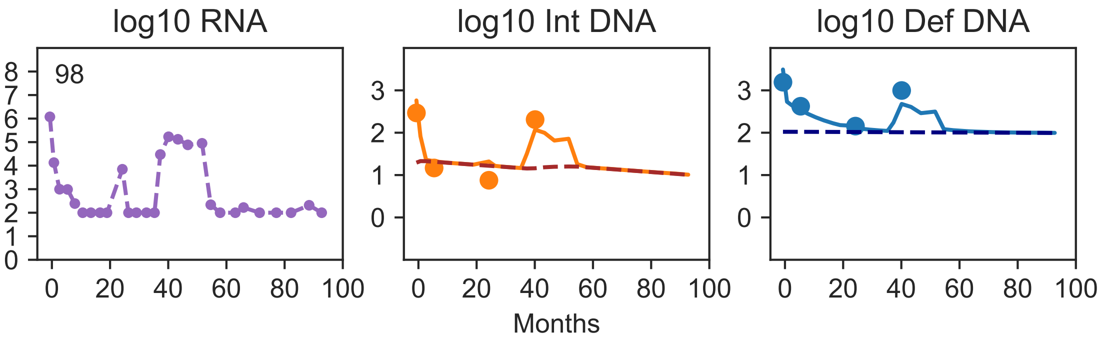

log10 RNA
log10 Int DNA
log10 Def DNA
98
Months
