# Supplementary material for: Intact HIV DNA decays in children with and without complete viral load suppression
Source: PLoS Pathog. 2025 Apr 4;21(4):e1013003. doi: 10.1371/journal.ppat.1013003 (PMC12002518; doi:10.1371/journal.ppat.1013003)
Supplement: Attachment — Submitted filename: reviewerresponse.docx [file ppat.1013003.s013.docx]

**We thank the referees for the positive response and very thoughtful comments and suggestions. We respond here in a point by point fashion with our responses in bold.**

Reviewer #1:

In this interesting study, Reeves et al. measured plasma HIV RNA levels, CD4+ T cell counts, and the levels of intact and defective HIV DNA proviruses using the cross-subtype intact proviral DNA assay in samples from 120 Kenyan children living with HIV (CWH). The study found that intact proviruses decayed faster than defective proviruses during both the first year of ART and over a longer period (years 1-8). Notably, in children who did not maintain viral suppression, modeling of HIV RNA and DNA revealed that although HIV DNA levels could transiently increase, intact proviral genomes were not significantly affected by brief treatment interruptions. This study provides valuable insights into the dynamics of intact and defective proviruses during the early years of life in children with HIV. It is particularly strong in its inclusion of data from children who did not remain virally suppressed, a group that represents a significant fraction of CWH. One of the most notable findings is the faster decay of intact HIV DNA compared to defective DNA in both the early (<1 year) and long-term (1-8 years) treatment periods. The authors also suggest that transient increases in HIV RNA, likely due to treatment interruptions, do not have a lasting impact on the long-term dynamics of the HIV reservoir. Overall, this study is well-conducted, and its findings are both novel and insightful. However, I have several suggestions that may enhance the clarity and impact of the manuscript:

**Thanks sincerely for the evident close reading and excellent ideas.**

Reviewer #2:

In this large study of 120 Kenyan children, the researchers examined the decay of intact proviruses in children who initiated ART in infancy between 1-12 months of age and maintaining different levels of virologic suppression. A cross-subtype intact proviral DNA assay was used to measure intact proviruses. The authors compared the decay of intact and defective proviruses between 55 children with perinatal HIV who had “durable suppression” and 65 who had non-suppressed viremia, supposedly due to ART-interruptions, although confirmation of non-suppressed viremia from ART interruption vs. intermittent ART is not verified. With the CS-IPDA, they found differential decay of intact and defectives with half-lives, with intact proviruses showing faster clearance than defective proviruses of 3 and 9 months, respectively. Ongoing decay of intact proviruses was found with a mean half-life of 22 months—while defective DNA showed no decay. The authors also went on to examine TCRβ clones in a subset of five children—where they discussed that CD4+ TCR β clones wax and waned in patterns more similar to cells with defective proviruses. Importantly, they report on despite ART interruptions and associations with increase in total HIV-1 DNA that intact proviral load did not increase significantly in children-, a finding that is relevant to the field of HIV remission and cure research for children where ART interruptions are required to detect remission.

Overall comments:

This is an important study that fills knowledge gaps on the decay dynamics of cell-associated HIV DNA in perinatal infections, with respect to intact and defective proviruses and as a function of states of virologic suppression. A state-of-the art assay—a cross-subtype Intact proviral HIV DNA assay was used, which the authors’ group has direct expertise. A well-characterized cohort of children are studied. The expertise on the perspectives from adult cohorts is also a study strength.

Overall. an important set of data to the field of pediatric HIV infection dynamics, but limited by the non-suppressed nature of the cohort, the use of a CS-IPDA with no provision on PPV and NPV on subtype A infection, and reporting on assay performance across 120 participants. The mathematical model on reservoir dynamics is flawed by the mere lack of suppression in the cohort since not all that is intact is likely to seed the reservoir.

**Thanks for this excellent and fair summary. We hope we have addressed some of your main points on assay validation, suppression criterion, and modeling in our responses to your specific critiques below.**

Critiques:

- The calculated half-lives of intact and defective proviruses and overall study findings need consideration for the limitations of the CS-IPDA, with respect with how the primers were inferred—from historic sequences in Los Alamos—I believe—
  - Perhaps the authors can provide any unpublished data on further validation of the CS-IPDA assay from direct analyses of proviral landscape single genome sequences on the cohort—in other words—some validation of the sensitivity and specificity for HIV subtype A , including positive and negative predictive value, as was done for other IPDA assays—Bruner et al., Bucholtz et al and Lee et al.

**We appreciate the concerns of this reviewer and have added a new section in the results on validating the CS-IPDA assay against the OPH cohort sequences. We only have single genome sequences for a few of these children, so we are unable to determine the complete PPV and PNV. However, we now present a similar analysis to Kinloch *et al* 2021. Of the 81 participants assessed by the CS-IPDA, only 5 (6.2%) and 10 of 356 total samples (3%) had no signal detection. Significantly, every CS-IPDA target was detected in all 81 individuals across sampled time points. Thus, the failure rate of our CS-IPDA is significantly lower than other IPDAs. We now present these results in a section Validating the CS-IPDA assay for OPH samples (l152)**

- In Figure 1—the suppressed subset—while with lower levels of viremia and area under the curve for viremia than the viremic subset, still appear to have ongoing viremia in the 2-3 log range over the time course of treatment, which poses a challenge for more precisely quantifying HIV reservoirs.

**Good point, we did endeavor to show that these occasional blips did not significantly raise HIV DNA (last version Supp Fig 3). However, based on yours and the other reviewer’s fair point, we performed an additional analysis (now supp figs 4 and 8) and added a new section to the results (l217). We changed the criteria to <300 and <1000 copies/mL and estimated intact HIV DNA decay rates in both of these groups, which resulted in a mean of ~20month and ~55month half-lives, in relatively good agreement with the mean value from the larger subset despite the wide uncertainty that arises from these low N estimates.**

- Additionally, within the first two years of ART, and with non-suppressed viremia, episomal HIV DNA can contributes to the total and intact proviral load.
  - Please provide an explanation for how the models account for changes in 2LTR circles in the first two years of ART

**Unfortunately, we did not quantify 2LTR circles. However, in prior studies in adults (White et al. PNAS 2022), the decay rates of 2LTR circles was similar to the decay of HIV DNA, so we believe not differentiating the 2LTR circles wouldn’t change our main results.**

- CD4+ T cell clonality data was done on a very small subset of the population—N=5, while these data are interesting—it seems insufficient to make meaning conclusions about HIV DNA kinetics
  - The conclusion—lines 308-309 seem strong---“We determined that that non--naïve CD4+ T cell clone kinetics could explain defective but not intact HIV DNA, suggesting intact proviruses influence the death rate of their carrier cells”.

**We recognize that in this case N=5 is small, but on the other hand, there were n~10k-100k clonotypes per person, and across all 5 individuals, the data are remarkably similar. Importantly, these are randomly chosen participants based on their data collection level, not their clinical characteristics. We believe the evidence is strong enough to say that cells harboring intact HIV DNA could not simply be behaving like most CD4+ T cells, but we did soften language in the discussion (l471)**

- The section on modeling transient rises in HIV DNA during RNA viremia is confusing to this reviewer as the deeply latent (l3) compartment in the model seems not applicable given the persistent viremic state of the participants; it is also unclear what the contribution of selected drug-resistant HIV will perform in this model. Hence the conclusion on page 8, lines 275 and 276 is unclear—“Importantly, model projections indicated that periods of viremia were strongly associated 276 with rises in 2 but not 3 cells (the more deeply latent reservoir)”—how is the deeply latent reservoir inferred here?

**This is a fair critique. In this modeling, we are essentially projecting that there is some small fraction of infected cells that eventually become the long-lived latent reservoir once the viremia is suppressed. We acknowledge that we cannot distinctly measure these populations. Based on this comment and others from Reviewer 2, we substantially rewrote the modeling sections to help with clarity.**

**Related to your other point, selected drug resistant mutations were not considered given that in all children ART resuppressed viremia. Unfortunately, we do not have enough data to see if predominant sequences evolved during these interruptions.**

**We added language to discussion (l547) that we cannot assess if drug resistance could be contributing to participant heterogeneity**

Reviewer #3:

The study by Reeves et al. Lehman addresses several important questions regarding CWH. The authors aptly point out to the “gold-standard” of HIV cure strategies involve ATI. Placed in the context of ATI in CWH, it is invaluable to understand intact HIV DNA dynamics in this population. The manuscript was well-written and thoughtful. Some specific comments follow, but most fall under the modeling.

**Thanks**

*Part II – Major Issues: Key Experiments Required for Acceptance. Please use this section to detail the key new experiments or modifications of existing experiments that should be absolutely required to validate study conclusions. Generally, there should be no more than 3 such required experiments or major modifications for a "Major Revision" recommendation. If more than 3 experiments are necessary to validate the study conclusions, then you are encouraged to recommend "Reject".*

Reviewer #1:

1. The definitions for “suppressed” and “viremic” participants are somewhat unclear. While I understand the post-stratification analysis presented in Figure S1, I believe it would be more appropriate to define viral suppression using a more stringent criterion, such as stratifying participants based on the area under the curve (AUC) of viral loads. Alternatively, how would the results change if a stricter definition of suppression were applied (e.g., <2 blips total, with viral loads <400 copies/mL)?

**Excellent point, we had originally made some versions with RNA AUC as viral load AUC during primary infection has been shown to associate with rebound timing. We now include this as a supplementary figure (Fig S4). Intriguingly, there isn’t a strong relationship between viral load AUC and long-term reservoir sizes – here we measure this as the last intact HIV DNA value. We think this supports our conclusion that much HIV DNA is harbored in short-lived infected cells**

**Based on this comment and from Reviewer 1 we also performed as sensitivity analysis where we adjusted our criteria. Similarly, there wasn’t a large difference in decay rate when we set viral load cutoff to 300, 1000, or our choice 3000 copies/mL (see new Supp Fig 8) but those sparser data sets did mean confidence intervals were wider.**

2. In several sections, the paper reads like a statistical analysis, but the biological implications and clinical relevance of the findings should be emphasized more. For instance, it is unclear why the authors compare “viral load decay from months 2-12 of ART” with “intact HIV DNA decay during year 0-1 of ART.” I recommend focusing on a smaller number of more meaningful comparisons. Additionally, correlations that did not reach statistical significance (e.g., Figure S6) should likely be removed, as they detract from the key findings.

**Ok, fair points. We have tried to rewrite to focus on biological interpretations and also to split those results section into one that is more about numerical values (to provide a clean reference on these values) and the second which emphasizes “differential decay”, the important conceptual observation for reservoir decay.**

**As it’s in the supplement, we do think it’s important to show all the associations we checked to be clear – also, some of these negative results are possibly useful to others.**

3. The analysis of cytokine correlations, particularly in Figure S7, may not be highly relevant to the core focus of the study. These correlations seem to dilute the more compelling associations observed between Granzyme B (GzB) and faster viral load decay, and between IL-15 and the faster decay of intact proviruses. Since these associations did not survive multiple comparison corrections, their inclusion may need reconsideration.

**We see your point but feel it is more honest to share all the comparisons. We were proud of our statistical rigor in this study design and we do want to report the negative results too. Both these decisions could help foster reproducible results in our field.**

4. The rationale for comparing the dynamics of intact and defective proviral DNA with TCR clonal dynamics (Figure 3F) is unclear. Given that clonal proliferation and contraction essentially balance out (i.e., homeostasis), and the observed decay in intact DNA (as reported previously), it is not surprising that these phenomena are largely independent. The statement that “defective DNA kinetics resemble CD4 count” (line 237) is not very conclusive and may be omitted.

**Though intuitive, we didn’t think this result had ever been shown, we actually think it’s very important to demonstrate that the building blocks of the reservoir (cd4+ T cell clones) cannot explain intact decay. Similarly on a bulk level, we think our results here can be used to make inference that defective HIV DNA is mostly a passenger on CD4+ T cell levels.**

5. The modeling portion of the study presents interesting conclusions but could benefit from clearer presentation. While the main conclusions outlined in lines 278-281 are understandable, the data supporting these statements should be presented more clearly. Figures 4C and D are difficult to interpret, and statistical support for the authors’ claims is needed. Was the modeling analysis necessary to demonstrate that short-term viremia does not permanently reseed the reservoir? Could this conclusion be drawn more simply by analyzing the longitudinal data already presented in the study?

**Thanks for this motivation. Based on this and other comments on the modeling, we substantially rewrote this section and reperformed all the modeling to help make more statistically rigorous comparisons. In particular we used the same exact model to estimate half lives for viremic and suppressed individuals to make comparisons more fair.**

**It might be possible to come to roughly the same conclusion from the raw data (i.e. new supp fig 4), but then again, this analysis is not highly convincing because there are so many complex variables (at minimum different numbers of time points and different timing of resuppression relative to measurements of DNA). Instead, we believe the modeling approach is an ideal one to immediately encapsulate the total amount of viremia and help mechanistically link to DNA.**

Reviewer #2: see above

**Addressed above**

Reviewer #3: None.

*Part III – Minor Issues: Editorial and Data Presentation Modifications*

*Please use this section for editorial suggestions as well as relatively minor modifications of existing data that would enhance clarity.*

Reviewer #1:

1. It would be helpful to place the results in context with other studies on the dynamics of HIV reservoir markers in the early years of life: Veldsman et al. AIDS 2018; Katussime et al. J Virol 2020; Massanella et al. Clin Infect Dis 2021. This would provide a clearer understanding of how the findings contribute to the broader field.

**Thanks, we tried to cite a broad range of the works on this important subject but apologize for missing these. Now added in intro and discussion references.**

2. Line 84: “CHW” should be corrected to “CWH.”

**Thanks.**

3. Line 221: “Repertoire sizes ranged from 4,904 to 61,288 sequences” – Is this referring to unique clonotypes or the total number of TCR sequences obtained?

**The total number, we have revised.**

4. Throughout the manuscript, the authors report HIV DNA levels per million T cells (i.e., CD4 and CD8). This is an unconventional method, and it would be helpful to clarify whether these measures were performed in PBMCs, T cells, or specifically in CD4+ T cells.

**In children, blood volumes (and therefore numbers of cells) collected are quite limited and thus we do not separate CD4 T cells from PBMCs to avoid cell loss during separation. However, our CS-IPDA includes ddPCR assays for both RPP30 to quantify the number of cells assayed and DeltaD, a host genomic region only found in non-T cells. These assays allow us to calculate the number of T cells tested in the CS-IPDA. Therefore, we are not able to quantify CD4+ T cells specifically. We now note this as a limitation in the discussion (l533) and also note that our decay rate estimates are not highly divergent from other published values, suggesting measurements per T cells isn’t introducing some large bias.**

5. In Figure S7 and the associated correlation matrices, it would be useful to include p-values to provide transparency on the statistical significance of the reported associations.

**Ok these are added, note figure is now S9**

6. The manuscript refers to “non-naïve cells.” It would be helpful to clarify whether this refers to CD45RA- or CD45RO+ cells, to ensure accuracy.

**Excellent point. The non-naïve cells are (mostly) CD45RO+. To be exact, they are PBMCs that are negative for: CD8a, CD14, CD15, CD16, CD19, CD36, CD56, CD123, TcRγ/δ, and CD235a AND positive for at least one of the following: CD25, CD45RO, and HLA-DR. So they might be CD45RO- but if so then they have either CD25 or HLA-DR as an activation marker. This is now included in the methods section on the sorting.**

7. Granzyme B is not technically a cytokine, but rather an effector molecule or cytotoxin. This distinction should be clarified.

**Thanks, we now adjusted the language throughout to “immune markers” and clarify these are interleukins and Granzyme B for our study.**

8. Line 345 mentions that the cells used for TCR sequencing were “resting” non-naïve T cells, but this contradicts the description in the methods section. The authors should reconcile these two statements to ensure consistency.

**Thanks for catching this. The non-naïve cells were not stimulated, but we didn’t sort out cells that were in an activated state already. We removed resting.**

Reviewer #2: see above

Reviewer #3:

1. While the authors offer evidence of what subtypes are predominantly found in study population, is there any sequenced based evidence to confirm that this is consistent. This would also ultimately support the use of CS-IPDA within the tested subtypes.

**Thanks for pointing this out. We now add data on this as Fig 1C. These are based on *pol* sequence for N=106 (88%) of the 120 children included here, with 68 (64.2%) living with subtype A, 10 (9.4%) subtype C, 21 (19.8%) subtype D, and 7 (6.6%) were recombinants. This is included in the first results section on participant information (line 113)**

**Moreover, see our response to reviewer 2 above, we now include a section showing very low failure rates to detect probes across all samples, validating CS-IPDA on these participant samples.**

2. Do the authors have plasma RNA (or DNA) sequence data from the suppressed subset where viremic episodes occurred, or in the viremic subset, to compare how and at what level, ongoing rounds of viral replication occurred? It would be interesting to consider the diversification of the virus since presumably the virus in plasma before ATI is what is observed in rebound viremia.

**We agree that it would be interesting to look at the rebound virus and evolution of the virus over time by sequencing during viremic episodes in this cohort. Unfortunately, we have only performed limited sanger sequencing on this cohort over time during select viremic episodes and would need funding to do more and deeper sequencing to answer this question and believe this is beyond the scope of this manuscript. However, we added a paragraph to the discussion to address this comment.**

3. The modeling methods are quite nice. In parts a bit of an oversimplification but it appears that the authors intended this to enjoy some relaxed fits within modeling the data. Some specific points:

**We appreciate this very nice summarization of the spirit of the modeling here.**

3A. Quite pedantic, but please define what “reservoir” and “latent reservoir” refer to in your study. I find by doing so lessens any squabbles. One can easily argue that the “reservoir” is that only of intact replication-competent proviruses. And by “latent”, referring to transcriptionally-silent or just all infected cells in the resting memory T cell phenotype? Personally, defective proviruses fall outside of the reservoir for this Reviewer, but in the context of the model and how the authors incorporate it, it is appropriate.

**We personally strongly agree with your point. The definition is important and we apologize for imprecision. We now state that the long-lived intacts are the key population which approximate a rebound competent reservoir. See (l372) and notation in the new Fig 4A. Moreover, we added substantially to the model methods to be more precise and specific about I2 and I3 being shorter and longer lived infected cells throughout.**

3B. Lines 529-548. Perhaps it can be supplemental, but the phrase “guesses” are interesting to use here. Did the authors use any a priori? How were the different models assessed—through progressive complexity and evaluated by AIC etc.? Why were the SDs assumed to be 1/10 mean?

**Thanks for calling this out. Given your fair comments, we have now updated the modeling to include less pre-specified assumptions, simply fitting all these parameters using pNLME based on the data and using a regressor for viral load. See the new methods section and results sections. We used published reservoir decay models (including our own which resemble those of many other well-known groups) rather than building a new one for these data though a couple other mathematical formulations were tried which are mentioned in the methods.**

3B.1. In general, the many assumptions and “guesses” could leave uninformed readers with some skepticism as to how the models were built and utilized. And ideas as the “passenger” hypothesis which (currently) does not include a reference that a reader could go back to, is something that needs to be defined.

**Thanks, this is a valuable suggestion. We hope our new formulation, which is built on more rigorous fitting methods and requires many fewer a priori assumptions/bounding of parameters would be less open to skepticism.**

**We now cite two of our published and pre-printed works that delves into why we use the term passenger hypothesis.**

3B.2. An overall suggestion is to use simplified jargon to support the decisions would be highly valuable and add definitions were appropriate.

**Thanks, we hope we’ve addressed your point by rewriting language associated with the viremia modeling section and introducing the concept of phases earlier on in the article.**
